# Stress Hyperglycemia Ratio and the Risk of Sepsis in Patients With Heart Failure: Retrospective Cohort Study From Medical Information Mart for Intensive Care-IV

**DOI:** 10.2196/81229

**Published:** 2026-04-17

**Authors:** Xianzhen Cai, Baoxin Yan, Jinhao Chen, Junjun Ye, Jiating Su, Shuangshuang Tong, Bin Xie, Bingna Zhang, Jilin Li

**Affiliations:** 1 Department of Cardiology, Second Affiliated Hospital of Shantou University Medical College, 69 Dongxia North Road, Shantou, China, 86 754 831411, 86 754 88346543; 2 Department of Cardiology, The Sixth People’s Hospital of Longgang District, Shenzhen, China

**Keywords:** stress hyperglycemia ratio, heart failure, sepsis, diabetes, mediation analysis

## Abstract

**Background:**

The occurrence of sepsis in patients with heart failure (HF) has received less attention in research; yet, it poses a significant clinical challenge due to the complex interplay between chronic cardiac dysfunction and acute systemic inflammation. The stress hyperglycemia ratio (SHR) has emerged as an independent risk factor in various cardiovascular diseases and patients with sepsis, but its role in predicting sepsis risk in patients with HF remains underexplored.

**Objective:**

This study investigates the association between SHR and sepsis occurrence in patients with HF and explores the potential mediating role of inflammatory indicators.

**Methods:**

This retrospective cohort study used data from the Medical Information Mart for Intensive Care-IV (version 3.0) database, encompassing patients with HF from critical care units. SHR was calculated based on initial blood glucose and glycated hemoglobin A_1c_ levels. The analysis population was divided into 4 groups based on the quartiles of the SHR. The primary end point was 7-day sepsis incidence, which was diagnosed following Sepsis-3 criteria.

**Results:**

Within the 1205-patient cohort (male: 764/1205, 63.4%; median 71.51, IQR 62.45-79.47), a total of 162 (13.4%) patients with HF experienced sepsis within 7 days. In the fully adjusted model, a per-unit SHR increase was linked to an 18% higher sepsis risk (hazard ratio 1.18, 95% CI 1.01‐1.38; *P*=.04). Restricted cubic splines analysis showed a nonlinear saturation effect association (*P* for nonlinearity=.02), which was consistent in the diabetic subgroup (*P* for nonlinearity=.01). After adjusting for 7-day mortality as a competing event using the Fine-Gray model, SHR was independently associated with an increased risk of sepsis (*P*=.01). The association between SHR and sepsis was significantly modified by diabetes mellitus, BMI, and insulin use (all *P* for interaction<.05). Furthermore, mediation analysis indicated that several inflammatory indices, including the systemic immune-inflammation index, neutrophil-to-lymphocyte ratio, platelet-to-neutrophil ratio, systemic inflammation response index, and monocyte-neutrophil-to-lymphocyte ratio, significantly mediated the association in critically ill patients with HF.

**Conclusions:**

In critically ill patients with HF, an elevated SHR was associated with heightened 7-day sepsis risk, especially for those combined with diabetes. Furthermore, systemic inflammatory indices partially mediated this association in the overall population, implicating inflammation as a potential mechanistic link between SHR and sepsis.

## Introduction

Heart failure (HF), a prevalent but serious disease, has seen a 29% global prevalence increase from 2010 to 2019 [1], with a 30%‐50% risk of mortality or rehospitalization within the acute phase [2].

The critically ill patients with HF are particularly vulnerable to sepsis, which may be related to immune dysfunction [3], cardiac remodeling [4], or bacterial translocation [5]. It was reported that the mortality rate reach 90% in those combined cardiac dysfunction and sepsis [6], and the recurrence rate of sepsis in patients with HF is 3-fold higher than those without HF [7].

Thus, identifying modifiable risk factors for sepsis occurrence is crucial for global public health and prevention strategies. The pathogenesis of sepsis in critical illness is multifactorial, with emerging evidence suggesting that hyperglycemia, especially under stress, may contribute to the development and prognosis of sepsis involving immune dysregulation, inflammatory activation, and organ impairment [8-10]. Stress hyperglycemia ratio (SHR) has emerged as a novel and potentially valuable metric in the assessment of acute glycemic levels, which is calculated by comparing admission blood glucose levels to glycated hemoglobin (HbA_1c_), distinguishing an acute stress-induced hyperglycemia from preexisting poor glycemic control [11]. Multiple studies have demonstrated that higher SHR levels were associated with poorer prognosis in patients diagnosed with acute coronary syndrome [12], acute HF [13], and sepsis [14]. However, the relationship between SHR and the occurrence of sepsis among patients with HF remains unclear. Inflammatory markers such as neutrophil-to-lymphocyte ratio (NLR), platelet-to-lymphocyte ratio, monocyte-to-lymphocyte ratio, neutrophil-to-platelet ratio (NPR), neutrophil-monocyte-to-lymphocyte ratio (NMLR), systemic immune-inflammation index (SII), and systemic inflammation response index (SIRI) have been investigated in patients with sepsis and show promise as prognostic indicators [15-20]. Given the intricate interplay between hyperglycemia and inflammation [21], and the well-documented role of inflammatory processes in the pathogenesis of sepsis, we hypothesize that inflammation may serve as a mediating factor in the association between SHR and sepsis occurrence. Therefore, this study aims to fill this critical gap by investigating the association of SHR and the risk of sepsis among critically ill patients with HF, based on data from the Medical Information Mart for Intensive Care-IV (MIMIC-IV) database. Furthermore, we explored whether inflammation acts as a potential mediator in this relationship.

## Methods

### Ethical Considerations

This study was a retrospective observational design with publicly available data from the MIMIC-IV database, which includes information on critically ill patients admitted to the intensive care units (ICUs) at Beth Israel Deaconess Medical Center from 2008 to 2022 [22]. The data acquisition process adhered to all relevant regulations. Author BY obtained a Collaborative Institutional Training Initiative license (Record ID 12861338) and the necessary permissions to extract data from the MIMIC-IV database. The project was approved by the institutional review boards of both the Massachusetts Institute of Technology and Beth Israel Deaconess Medical Center and followed the Strengthening the Reporting of Cohort Studies in Surgery guidelines [23]. Our research was conducted in accordance with the Declaration of Helsinki. Since the MIMIC-IV database includes fully anonymous or deidentified patient records and all protected health information has been removed in accordance with Health Insurance Portability and Accountability Act (HIPAA) privacy standards, participants or the participants’ legal guardians or next of kin consent for publication is not applicable to this study. This study did not involve any ethical conflict issues.

### Study Design and Participants

**Figure 1.**
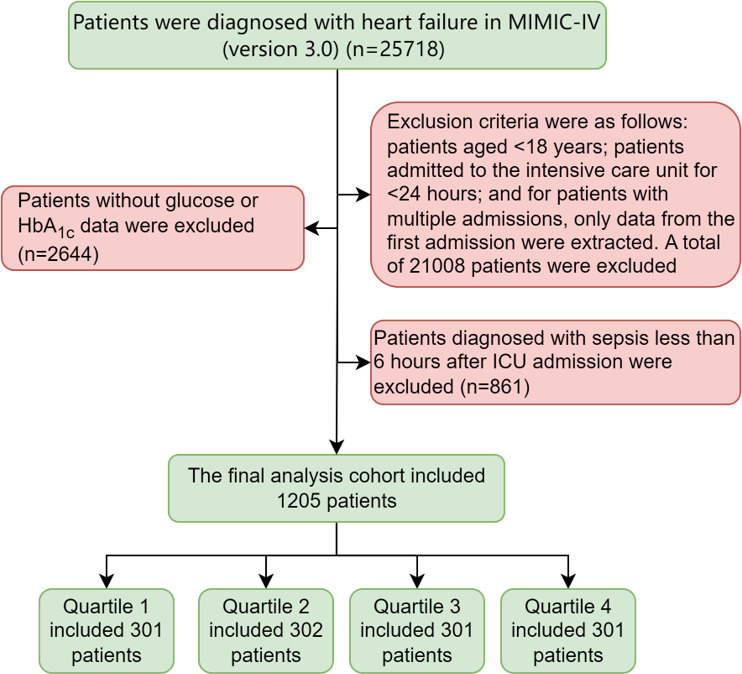
Patient selection flowchart. Flowchart illustrating the inclusion and exclusion criteria for patient selection in a retrospective cohort study investigating the association between the stress hyperglycemia ratio and 7-day sepsis risk among critically ill patients with heart failure. Data were derived from the MIMIC-IV database, Beth Israel Deaconess Medical Center (Boston, MA, United States), encompassing admissions from 2008 to 2022. A total of 1205 patients were included in the final analysis. Inclusion and exclusion criteria are detailed. HbA_1c_: glycated hemoglobin; ICU: intensive care unit; MIMIC-IV: Medical Information Mart for Intensive Care-IV.

This is a retrospective observational study based on a large-scale critical care database, which included the ICU patients who were diagnosed with HF based on the *International Classification of Diseases, Ninth Revision* (ICD-9) and *International Classification of Diseases, Tenth Revision* (ICD-10) codes (Table S1 in Multimedia Appendix 1). We excluded (1) patients younger than 18 years of age; (2) patients with an ICU stay duration less than 24 hours; (3) for patients with multiple ICU admissions, only data from the first hospitalization were included; (4) patients lacked data of blood glucose or HbA_1c_; and (5) patients diagnosed with sepsis [24] less than 6 hours after ICU admission. Finally, a total of 1205 patients were enrolled in the study cohort and divided into 4 groups based on SHR quartile (Figure 1). Quartile 4 representing the highest SHR was consistent with previously published studies [25].

### Definition of Sepsis

Sepsis was defined according to Sepsis-3 criteria [3]. The time of sepsis onset (*T*_sepsis_) was determined as the earlier of the first clinical suspicion of infection (*T*_suspicion_) and the first time the Sequential Organ Failure Assessment (SOFA) score increased by ≥2 points (*T*_SOFA_), provided that *T*_SOFA_ occurred no more than 24 hours before or 12 hours after *T*_suspicion_ according to published literature [24]. To specifically capture new-onset sepsis developing during the ICU stay and to minimize the inclusion of infections likely present at admission, we excluded all patients for whom *T*_sepsis_ occurred within the first 6 hours after ICU admission in our primary analysis.

### Inflammation Biomarkers

Inflammatory biomarkers included NLR, platelet-to-lymphocyte ratio, monocyte-to-lymphocyte ratio, NPR, NMLR, SII, and SIRI. Detailed calculation methods are provided in Table S2 in Multimedia Appendix 1.

### Data Collection

The SHR was calculated using the formula: SHR=admission blood glucose (mg/dL)/ (28.7×HbA_1c_ (%)−46.7) [11]. Blood glucose and HbA_1c_ values were sourced from the initial records after ICU admission. The baseline variables were chosen on the bases of their possible influence on sepsis risk and cardiovascular risk of the individuals and followed six categories: (1) demographics (sex, age, race, and BMI), (2) vital signs (heart rate, systolic blood pressure, diastolic blood pressure, mean blood pressure, respiratory rate, temperature, and 6-hour fluid intake), (3) laboratory parameters (hemoglobin, white blood cell, platelet, blood urea nitrogen, creatinine, glucose, HbA_1c_, etc), (4) comorbidities (diabetes, hypertension, shock, etc), (5) disease severity scores (Charlson comorbidity index, Acute Physiology Score III, systemic inflammatory response syndrome score, SOFA score, etc), and (6) medication (angiotensin-converting enzyme inhibitors/angiotensin receptor blockers, diuretics, antibiotics drugs, insulin, glucocorticoid, oral hypoglycemic drugs, etc). For all baseline variables, the first recorded value within the initial 6 hours after ICU admission was extracted using PostgreSQL (version 13.7.1; PostgreSQL Global Development Group) and Navicat Premium (version 17; PremiumSoft CyberTech Ltd) and used in the analysis. This study adhered to the STROBE (Strengthening the Reporting of Observational Studies in Epidemiology) guidelines for observational studies [26]. Variables with a missing rate ≤25% were imputed using the Multivariate Imputation by Chained Equations method, generating 20 imputed datasets, applying predictive mean matching for continuous variables and logistic regression for categorical variables. The imputation model included all baseline variables used in the analysis. Variables with a missing rate >25% were excluded to avoid bias. Further details are provided in Table S3 in Multimedia Appendix 1.

### Definition of Outcomes

The primary outcome was the occurrence of sepsis within 7 days after ICU admission, which was defined based on the acute disease distribution patterns observed in our dataset (Figure S1 in Multimedia Appendix 1) and was consistent with the early prediction window commonly adopted in prior studies focusing on acute sepsis in ICU settings [27]. Secondary outcomes included 7-day mortality, a composite outcome of sepsis occurrence or mortality within 7 days, 28-day mortality, and in-hospital mortality.

### Statistical Analysis

Continuous variables were described using median (IQR) or mean (SD), and comparisons between groups were performed using the Kruskal-Wallis test or ANOVA, as appropriate. Whereas categorical variables were expressed as frequency (%) and were analyzed using the Fisher exact test or the Pearson chi-square test. The Kaplan-Meier survival analysis was used to estimate the occurrence of primary outcomes among groups according to the log-rank test. To assess the impact of SHR on sepsis occurrence, the multivariate proportional hazard regression models (Cox regression models) were used to assess the hazard ratio (HR) and 95% CI for event occurrence. Specifically, model 1 was unadjusted for covariates, and model 2 further adjusted sex, age, and BMI. Considering the impacts of patient general condition, vital signs, SOFA scores, and steroid use on outcomes, based on model 2, model 3 incorporated acute HF, diabetes, shock, SOFA score, antibiotic, insulin, and glucocorticoid. To explore the potential dose-response relationship between SHR and the risk of sepsis occurrence, restricted cubic splines (RCS) analysis was performed and adjusted by the same models in model 3. Furthermore, subgroup analyses were conducted to delve deeper into the data, stratifying outcomes based on sex, age, BMI, diabetes, acute HF, and insulin use. These subgroup analyses were performed using comprehensive regression models adjusted for potential confounding factors. To test whether diabetes status modifies the relationship between SHR and sepsis risk, we introduced an interaction term between SHR (as a continuous variable) and diabetes status (yes or no) within the Cox regression model, which was also adjusted for the same covariates as in the fully adjusted model (model 3). Moreover, to rigorously evaluate the robustness of our main findings, sensitivity analyses were performed by logistic regression, Fine-Gray, and competing risk Cox models. In addition, we extended the exclusion window for incident sepsis from the original 6 hours to 12, 24, 36, and 48 hours after ICU admission, respectively, and repeated the Cox proportional hazards regression models.

To determine whether inflammatory indices (such as NLR and SII) mediate the association between SHR and sepsis occurrence, we used the Cox model in the *CMAverse* package in R (R Foundation for Statistical Computing). We performed 1000 bootstrap simulations to estimate each mediator’s effect and calculate the mediation proportion. The average direct effect represents the impact of SHR on sepsis without mediation, while the average causal mediation effect indicates the effect of SHR on sepsis occurrence via mediators. The mediation proportion was calculated by dividing the average causal mediation effect by the total effect. All statistical analyses were executed using R software (version 4.4.1), with statistical significance set as a 2-sided *P* value of less than .05.

## Results

### Baseline Characteristics

A total of 25,718 patients with HF from the ICU were consecutively recruited. Following certain exclusion criteria, 1205 patients were ultimately included, of whom 63.4% (n=764) were male, and had a median age of 71.51 (IQR 62.45‐79.47) years. Table 1 presents the baseline characteristics. There were 297 (24.6%) patients with hypertension, 564 (46.8%) patients with diabetes, 855 (71%) patients with acute HF, and 595 (49.4%) patients with pneumonia.

**Table 1.** Baseline characteristics of study participants stratified by stress hyperglycemia ratio (SHR) quartiles^a^.

	Overall (N=1205)	Groups of SHR^b^				*P* value
		Q1 (n=301)	Q2 (n=302)	Q3 (n=301)	Q4 (n=301)	
Male, n (%)	764 (63.4)	206 (68.4)	194 (64.2)	188 (62.5)	176 (58.5)	.08
Age (years), median (IQR)	71.51 (62.45‐79.47)	71.64 (60.73‐79.40)	71.69 (62.35‐79.97)	71.61 (62.25‐79.25)	71.15 (63.13‐79.61)	.92
Race, n (%)						.38
Black	108 (9)	26 (8.6)	30 (9.9)	21 (7)	31 (10.3)	
White	800 (66.4)	202 (67.1)	206 (68.2)	207 (68.8)	185 (61.5)	
Others	297 (24.6)	73 (24.3)	66 (21.9)	73 (24.3)	85 (28.2)	
BMI (kg/m^2^), median (IQR)	28.54 (24.42‐32.92)	28.73 (25.10‐33.59)	27.94 (24.42‐31.99)	28.64 (24.54‐32.87)	28.20 (23.93‐33.20)	.65
HbA_1c_^c^ (%), median (IQR)	6.00 (5.60‐7.10)	6.50 (5.90‐8.00)	5.80 (5.50‐6.57)	5.90 (5.50‐6.70)	6.00 (5.50‐7.40)	<.001
Glucose (mmol/L), median (IQR)	133.00 (106.00‐182.00)	99.00 (89.00‐115.00)	115.00 (104.00‐134.75)	140.00 (123.00‐171.00)	219.00 (175.00‐275.00)	<.001
SHR, median (IQR)	1.03 (0.85‐1.32)	0.74 (0.62‐0.80)	0.94 (0.90‐0.99)	1.13 (1.08‐1.21)	1.59 (1.42‐1.86)	<.001
Vital signs, median (IQR)						
Heart rate (bpm)	81.28 (73.59‐91.48)	81.04 (73.41‐89.58)	80.85 (74.38‐89.13)	81.54 (74.40‐93.11)	82.31 (72.71‐92.97)	.43
SBP^d^ (mm Hg)	111.48 (104.13‐122.04)	109.90 (104.39‐119.23)	111.00 (105.26‐119.29)	111.70 (103.58‐122.68)	113.79 (104.18‐126.50)	.08
DBP^e^ (mm Hg)	60.85 (53.94‐69.63)	58.71 (52.62‐65.57)	59.06 (52.74‐67.47)	62.10 (55.45‐70.69)	64.44 (56.58‐72.86)	<.001
MBP^f^ (mm Hg)	75.91 (70.45‐83.49)	74.24 (69.61‐81.09)	74.66 (70.02‐81.41)	77.53 (70.95‐84.12)	78.42 (71.95‐86.63)	<.001
Respiratory rate (breaths per minute)	19.08 (17.22‐21.06)	18.65 (16.91‐20.48)	18.87 (17.01‐20.92)	19.36 (17.29‐21.31)	19.50 (17.67‐21.61)	<.001
Temperature (℃)	37.06 (36.83‐37.44)	37.06 (36.83‐37.40)	37.10 (36.89‐37.50)	37.11 (36.83‐37.44)	37.06 (36.83‐37.50)	.54
Intake (mL per 6 hours)	499.86 (227.77‐1221.86)	579.12 (240.00‐1275.58)	718.86 (254.32‐1654.32)	477.99 (244.53‐1111.45)	382.35 (165.92‐800.00)	<.001
Laboratory test, median (IQR)						
Hemoglobin (g/dL)	12.00 (10.40‐13.70)	11.60 (9.80‐13.40)	12.00 (10.70‐13.47)	12.30 (10.50‐13.90)	12.20 (10.40‐13.80)	.03
White blood cell (10^9^/L)	12.10 (8.80‐16.60)	11.20 (8.00‐16.50)	12.60 (8.70‐17.78)	11.90 (9.20‐15.50)	12.30 (9.40‐16.60)	.05
Platelets (10^9^/L)	217.00 (176.00‐274.00)	207.00 (171.00‐262.00)	214.50 (171.50‐267.75)	210.00 (174.00‐265.00)	234.00 (189.00‐287.00)	<.001
HCT^g^ (%)	36.80 (31.90‐41.20)	35.60 (30.90‐41.00)	36.75 (32.40‐41.18)	37.70 (32.10‐41.80)	37.00 (32.00‐41.40)	.22
BUN^h^ (mg/dL)	24.00 (18.00‐36.00)	25.00 (18.00‐38.00)	22.00 (17.00‐33.00)	23.00 (17.00‐33.00)	25.00 (19.00‐43.00)	.001
Creatinine (μmol/L)	1.20 (0.90‐1.60)	1.20 (0.90‐1.70)	1.20 (0.90‐1.58)	1.10 (0.90‐1.50)	1.20 (1.00‐1.80)	.005
Chloride (mmol/L)	104.00 (100.00‐107.00)	104.00 (101.00‐107.00)	105.00 (101.00‐108.00)	103.00 (99.00‐106.00)	102.00 (98.00‐105.00)	<.001
Sodium (mmol/L)	139.00 (136.00‐141.00)	139.00 (137.00‐141.00)	139.00 (137.00‐141.00)	138.00 (136.00‐141.00)	138.00 (135.00‐141.00)	<.001
Potassium (mmol/L)	4.40 (4.00‐4.80)	4.40 (4.00‐4.70)	4.30 (4.00‐4.70)	4.40 (4.00‐4.70)	4.50 (4.10‐5.00)	.004
Bicarbonate (mmol/L)	25.00 (22.00‐27.00)	25.00 (23.00‐28.00)	25.00 (23.00‐27.00)	24.00 (22.00‐27.00)	23.00 (21.00‐26.00)	<.001
PT^i^ (seconds)	14.70 (12.90‐17.40)	15.30 (13.50‐17.90)	15.70 (13.60‐18.00)	14.30 (12.70‐17.40)	13.70 (12.40‐16.40)	<.001
APTT^j^ (seconds)	41.50 (30.90‐73.80)	40.50 (31.60‐72.40)	41.45 (31.63‐72.57)	41.30 (30.20‐74.00)	43.80 (29.10‐79.90)	.94
Comorbidities, n (%)						
Acute HF^k^	855 (71)	205 (68.1)	210 (69.5)	209 (69.4)	231 (76.7)	.08
Hypertension	297 (24.6)	73 (24.3)	83 (27.5)	73 (24.3)	68 (22.6)	.56
Coronary heart disease	934 (77.5)	229 (76.1)	220 (72.8)	234 (77.7)	251 (83.4)	.02
AMI^l^	551 (45.7)	142 (47.2)	144 (47.7)	131 (43.5)	134 (44.5)	.69
Valve disorder	540 (44.8)	166 (55.1)	146 (48.3)	122 (40.5)	106 (35.2)	<.001
Atrial fibrillation	604 (50.1)	163 (54.2)	151 (50)	157 (52.2)	133 (44.2)	.08
COPD^m^	329 (27.3)	99 (32.9)	84 (27.8)	73 (24.3)	73 (24.3)	.06
Chronic kidney disease	421 (34.9)	118 (39.2)	103 (34.1)	95 (31.6)	105 (34.9)	.26
Liver disease	74 (6.1)	23 (7.6)	22 (7.3)	19 (6.3)	10 (3.3)	.11
Cancer	40 (3.3)	5 (1.7)	8 (2.6)	16 (5.3)	11 (3.7)	.08
Diabetes	564 (46.8)	166 (55.1)	100 (33.1)	138 (45.8)	160 (53.2)	<.001
Lipoprotein metabolism disorders	800 (66.4)	216 (71.8)	203 (67.2)	186 (61.8)	195 (64.8)	.07
Shock	284 (23.6)	81 (26.9)	58 (19.2)	60 (19.9)	85 (28.2)	.01
Pneumonia	104 (8.6)	28 (9.3)	25 (8.3)	26 (8.6)	25 (8.3)	.97
Disease severity score, median (IQR)						
Charlson comorbidity index	6.00 (4.00‐8.00)	6.00 (5.00‐8.00)	6.00 (4.00‐8.00)	6.00 (4.00‐8.00)	7.00 (5.00‐8.00)	.05
APSIII^n^	40.00 (31.00‐50.00)	41.00 (32.00‐53.00)	39.00 (30.00‐48.75)	38.00 (31.00‐49.00)	41.00 (32.00‐52.00)	.15
SAPSII^o^	35.00 (28.00‐43.00)	35.00 (29.00‐43.00)	37.00 (31.00‐44.00)	34.00 (26.00‐43.00)	35.00 (28.00‐42.00)	.05
SIRS^p^	3.00 (2.00‐3.00)	3.00 (2.00‐3.00)	3.00 (2.00‐3.00)	3.00 (2.00‐3.00)	3.00 (2.00‐3.00)	.49
SOFA^q^	3.00 (1.00‐5.00)	4.00 (2.00‐6.00)	3.00 (1.00‐6.00)	2.00 (1.00‐4.00)	2.00 (1.00‐4.00)	<.001
Medication at baseline, n (%)						
ACEI/ARB^r^	398 (33)	108 (35.9)	106 (35.1)	95 (31.6)	89 (29.6)	.31
Amiodarone	154 (12.8)	37 (12.3)	38 (12.6)	33 (11)	46 (15.3)	.45
Anticoagulants	139 (11.5)	48 (15.9)	29 (9.6)	32 (10.6)	30 (10)	.05
NSAID^s^	770 (63.9)	210 (69.8)	202 (66.9)	195 (64.8)	163 (54.2)	<.001
β-Blocker	737 (61.2)	203 (67.4)	209 (69.2)	174 (57.8)	151 (50.2)	<.001
Diuretic drug	751 (62.3)	197 (65.4)	204 (67.5)	177 (58.8)	173 (57.5)	.03
Hypolipidemic drug	751 (62.3)	206 (68.4)	194 (64.2)	187 (62.1)	164 (54.5)	.004
Antibiotics	595 (49.4)	184 (61.1)	193 (63.9)	130 (43.2)	88 (29.2)	<.001
Insulin	747 (62)	221 (73.4)	192 (63.6)	163 (54.2)	171 (56.8)	<.001
Glucocorticoid	167 (13.9)	49 (16.3)	50 (16.6)	34 (11.3)	34 (11.3)	.09
Oral hypoglycemic drugs	19 (1.6)	8 (2.7)	5 (1.7)	1 (0.3)	5 (1.7)	.15

a Baseline demographic, clinical, laboratory, and treatment characteristics of 1205 critically ill patients with HF, stratified by quartiles of the stress hyperglycemia ratio. Data were derived from the Medical Information Mart for Intensive Care-IV database (Boston, MA, United States; 2008‐2022). *P* values were calculated using the Kruskal-Wallis test for continuous variables and the Pearson chi-square or Fisher exact test for categorical variables.

b SHR quartiles: Q1: <0.8490; Q2: 0.8490-1.0331; Q3: 1.0331-1.3177; Q4: >1.3177.

c HbA_1c_: glycated hemoglobin.

d SBP: systolic blood pressure.

e DBP: diastolic blood pressure.

f MBP: mean blood pressure.

g HCT: hematocrit.

h BUN: blood urea nitrogen.

i PT: prothrombin time.

j APTT: activated partial thromboplastin time.

k HF: heart failure.

l AMI: acute myocardial infarction.

m COPD: chronic obstructive pulmonary disease.

n APSIII: Acute Physiology Score III.

o SAPSII: Simplified Acute Physiology Score II.

p SIRS: systemic inflammatory response syndrome.

q SOFA: Sequential Organ Failure Assessment.

r ACEI/ARB: angiotensin-converting enzyme inhibitor/angiotensin receptor blocker.

s NSAID: nonsteroidal anti-inflammatory drug.

All participants were stratified into 4 groups (Q1-4) based on SHR quartiles: Q1: <0.8490 (n=301); Q2: 0.8490‐1.0331 (n=302); Q3: 1.0331‐1.3177 (n=301); and Q4: >1.3177 (n=301). The highest SHR group (Q4) exhibited increased vital signs, including respiratory rate and diastolic blood pressure, alongside elevated laboratory values such as platelet count and serum potassium levels. They had a higher burden of comorbidities (eg, coronary heart disease) and disease severity scores (Charlson index and Simplified Acute Physiology Score II), but lower antibiotic use. In contrast, the lowest SHR group (Q1) had higher HbA_1c_, diabetes prevalence, and insulin use. However, parameters such as age, BMI, and race levels showed no significant variation across SHR groups.

Furthermore, for clinical outcome, Table S4 in Multimedia Appendix 1 provides a comparison of baseline characteristics between patients with sepsis and those without sepsis within 7 days after admitted to the ICU. Compared to patients without sepsis, those with sepsis exhibited significantly elevated levels of SHR (median 1.13, IQR 0.92‐1.46 vs median 1.02, IQR 0.84‐1.29; *P*=.001) and higher inflammation infection.

Considering SHR be associated with diabetes status, compared to patients without diabetes, those with diabetes had higher BMI (28.96 vs 27.90 kg/m²) and no significant difference in SHR (*P*=.79; Table S5 in Multimedia Appendix 1).

### Relationship Between SHR and Parameters Related to Sepsis Diagnosis

Figures S2 and S3 in Multimedia Appendix 1 illustrate the relationship between SHR and markers of sepsis. The association between SHR and antibiotic use (defined as administration during the entire ICU stay) was assessed using the point-biserial correlation coefficient, yielding a weak negative correlation (*r*=−0.196). The Mann-Whitney *U* test further confirmed a difference (*P*<.001). In contrast, the linear relationship between SHR and SOFA score was evaluated using Spearman correlation and was not statistically significant (*r*=0.056; *P*=.05).

### Clinical Outcomes According to SHR Levels

In this study, sepsis occurred in 162 (13.4%) patients with HF, among whom, the Q4 (SHR >1.3177) exhibited the highest sepsis occurrence at 17.3%, thus being chosen as the reference group, and Q1 to Q4 showed progressively higher risks (9.6% vs 11.3% vs 15.6% vs 17.3%). Notably, the Q4 group exhibited a significantly higher mortality rate of 17.3% (52/301) and a composite outcome rate of 34.2% (103/301) compared with all other groups (both *P*<.001; S6 in Multimedia Appendix 1).

The Kaplan-Meier curves illustrated that patients with the highest SHR (Q4) exhibited the highest 7-day all-cause mortality across different SHR levels (*P*=.02; log-rank *P*<.001; Figure S4 in Multimedia Appendix 1). Specifically, although the association was nonsignificant in the diabetes subgroup (*P*=.17) and the nondiabetes subgroup (*P*=.07), the Q4 had relatively the highest 7-day all-cause mortality in the diabetes subgroup, while in the nondiabetes subgroup, patients in Q3 and Q4 groups had higher 7-day all-cause mortality than other groups (Figures S5 and S6 in Multimedia Appendix 1).

### Association of SHR Index With Sepsis Occurrence

To explore the independent relationship of SHR index with the occurrence of sepsis, we constructed 3 Cox regression models (Table 2), and significant values were all found in models 1‐3. When SHR emerged as a continuous variable, in the fully adjusted model 3, per 1-unit higher SHR index was associated with an 18% higher risk of sepsis occurrence (HR 1.18, 95% CI 1.01‐1.38; *P*=.04). Additionally, similar trends were observed when patients were grouped according to quartiles of the SHR index. Taking the highest quartile of the SHR index (Q4) as the reference, the occurrence risk of sepsis in other groups showed a gradual upward trend of Q1 <Q2 <Q4 (HR 0.52, 95% CI 0.32‐0.82; *P*=.005 vs HR 0.62, 95% CI 0.40‐0.97; *P*=.04), while compared to Q4, Q3 showed no significant difference (HR 0.96, 95% CI 0.65‐1.43; *P*=.85), indicating a dose-response relationship between SHR levels and sepsis risk. However, the trend test indicated a significant upward trend in sepsis risk with increasing quartiles (*P* for trends=.001).

**Table 2.** Cox regression models for the association between stress hyperglycemia ratio (SHR) and 7-day sepsis occurrence^a^.

	Events, n	Cases, n	Model 1^b^		Model 2^c^		Model 3^d^	
			HR^e^ (95% CI)	*P* value	HR (95% CI)	*P* value	HR (95% CI)	*P* value
SHR	—^f^	—	1.31 (1.11‐1.54)	.002	1.34 (1.13‐1.59)	.001	1.18 (1.01‐1.38)	.04
Q1	29	301	0.54 (0.34‐0.84)	.007	0.54 (0.34‐0.85)	.008	0.52 (0.32‐0.82)	.005
Q2	34	302	0.62 (0.40‐0.96)	.03	0.62 (0.40‐0.96)	.03	0.62 (0.40‐0.97)	.04
Q3	47	301	0.90 (0.61‐1.33)	.59	0.90 (0.60‐1.33)	.59	0.96 (0.65‐1.43)	.85
Q4	52	301	Reference	—	Reference	—	Reference	—
*P* for trends	—	—	1.25 (1.09‐1.44)	.002	1.25 (1.08‐1.44)	.002	1.26 (1.09‐1.46)	.001

a Multivariable Cox proportional hazards regression model evaluating the association between the SHR (both as a continuous variable and by quartiles) and the risk of sepsis within 7 days of intensive care unit admission among 1205 critically ill patients with heart failure. Data were derived from the Medical Information Mart for Intensive Care-IV database (Boston, MA, United States; 2008‐2022).

b Model 1: unadjusted.

c Model 2: adjusted for sex, age, and BMI.

d Model 3: adjusted for sex, age, BMI, acute heart failure, diabetes, shock, Sequential Organ Failure Assessment score, antibiotic use, insulin use, and glucocorticoid use.

e HR: hazard ratio.

f Not available.

The interaction analysis between SHR and diabetes was statistically significant (HR 1.96, 95% CI 1.23‐3.14; *P*=.005), indicating that the association between SHR and sepsis was stronger in patients with diabetes. Similarly, a significant interaction was also observed with insulin use (HR 0.48, 95% CI 0.30‐0.77; *P*=.002), whereas interactions with BMI (both continuous and categorized) were not statistically significant (*P*=.17 and *P*=.07, respectively; Table S7 in Multimedia Appendix 1).

**Figure 2.**
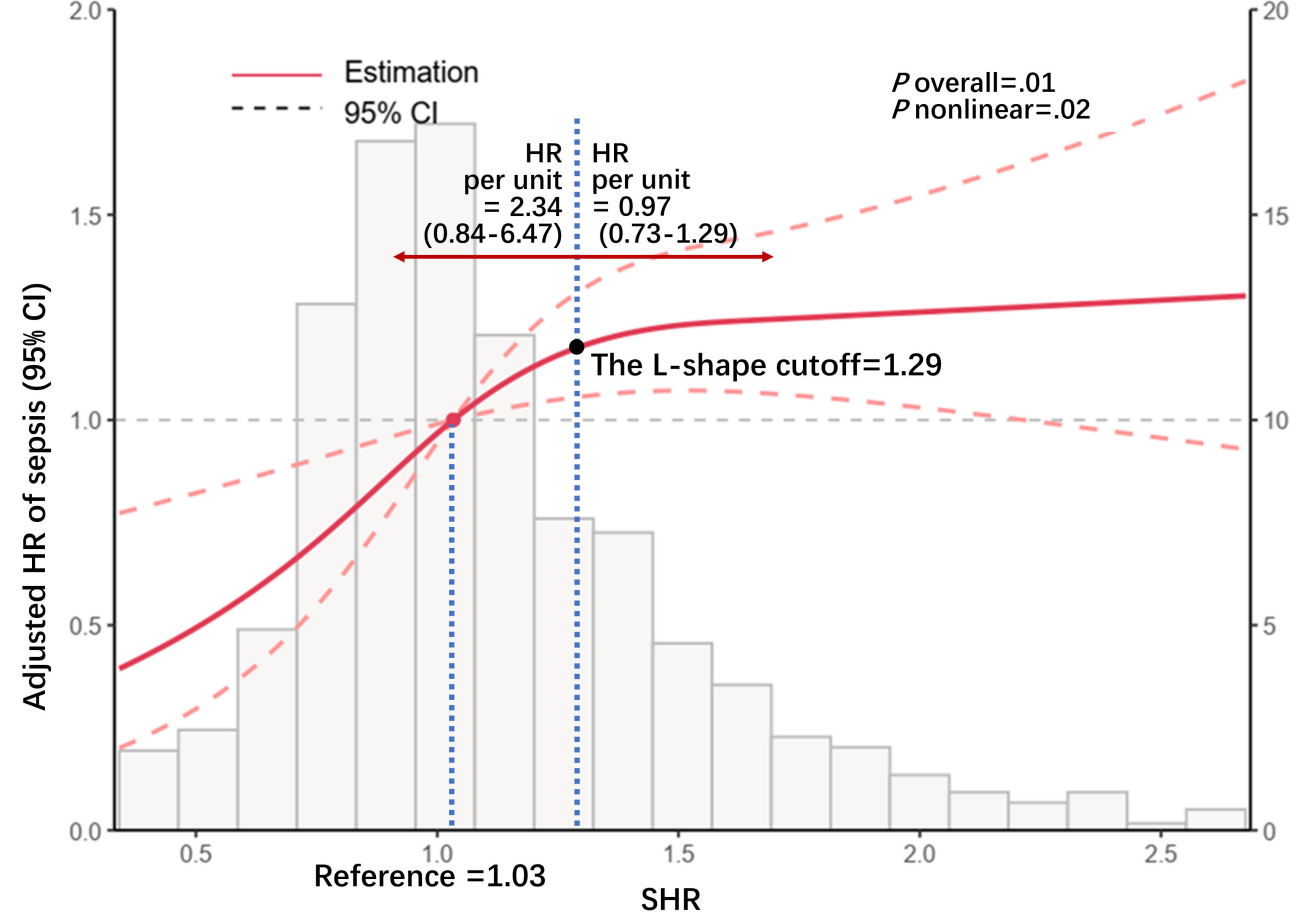
Restricted cubic spline analysis of the association between SHR and 7-day sepsis risk. Restricted cubic spline curve depicting the dose-response relationship between the SHR (as a continuous variable) and the adjusted HR for 7-day sepsis occurrence among 1205 critically ill patients with heart failure. Data were derived from the MIMIC-IV database (Boston, MA, United States; 2008‐2022). The model was adjusted for sex, age, BMI, acute heart failure, diabetes, shock, Sequential Organ Failure Assessment score, antibiotic use, insulin use, and glucocorticoid use. The curve demonstrates a nonlinear saturation effect, with an inflection point at SHR=1.29 (*P* for nonlinearity=.02). The horizontal dashed line represents an HR of 1.0. The shaded area represents the 95% CI. HR: hazard ratio; MIMIC-IV: Medical Information Mart for Intensive Care-IV; SHR: stress hyperglycemia ratio.

Additionally, multivariable adjusted RCS analysis was used to provide a comprehensive examination of the continuous relationship between SHR and the occurrence of sepsis. Relationship showed a nonlinear saturation effect as SHR increased, with the specific turning point of 1.29 (*P* for nonlinearity=.02; Figure 2). Specifically, sepsis risk increased steeply with SHR up to 1.29, after which the risk curve plateaued.

### Subgroup Analysis

**Figure 3.**
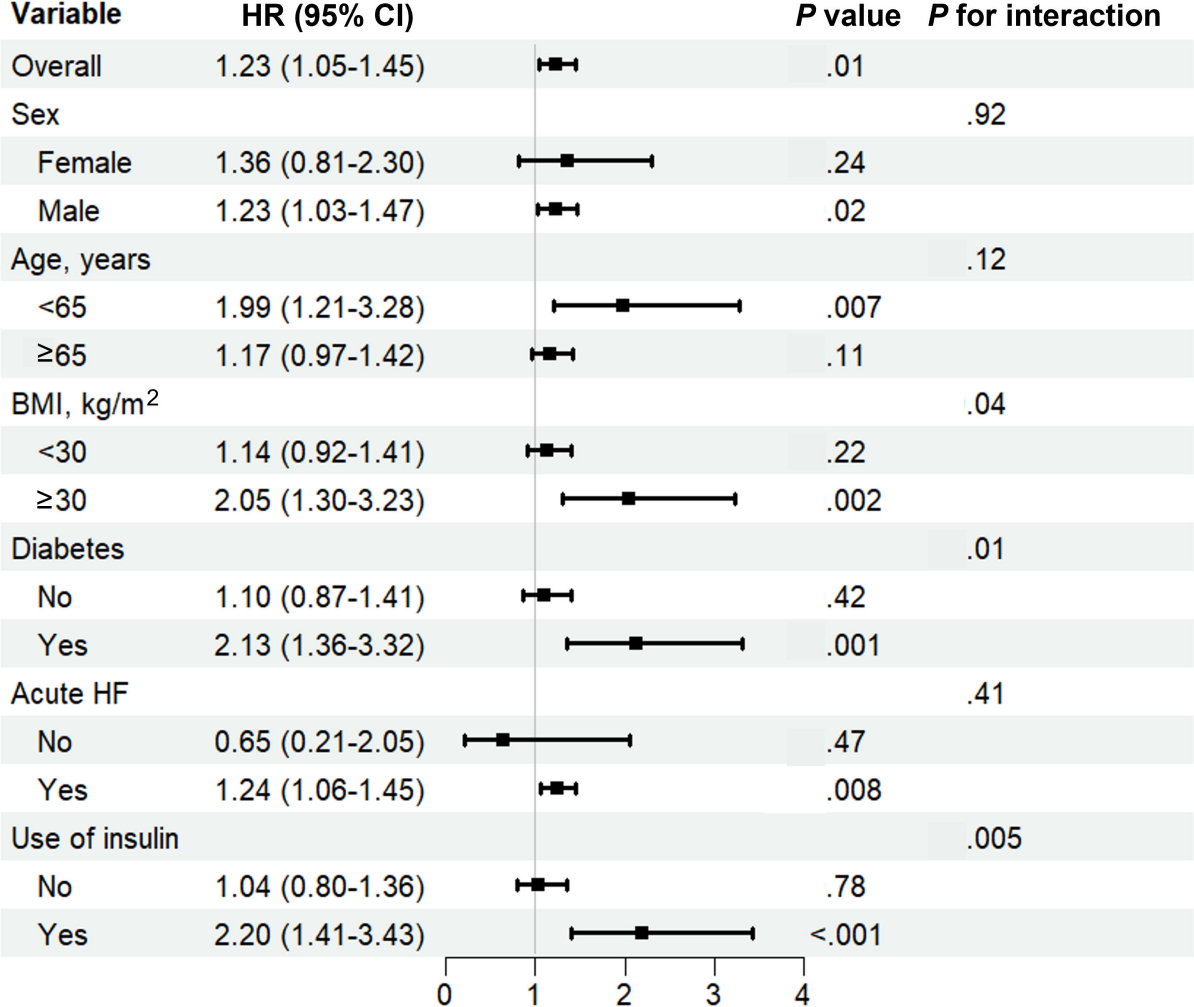
Subgroup analysis forest plot. Forest plot summarizing subgroup analyses of the association between the stress hyperglycemia ratio (as a continuous variable) and 7-day sepsis risk among 1205 critically ill patients with HF. Data were derived from the MIMIC-IV database (Boston, MA, United States; 2008‐2022). Subgroups were stratified by sex, age (<65 vs ≥65 years), BMI (<30 vs ≥30 kg/m^2^), diabetes status, acute HF, and insulin use. HRs and 95% CIs were adjusted for sex, age, BMI, acute HF, diabetes, and insulin use. *P* values for interaction are displayed to assess effect modification. HF: heart failure; HR: hazard ratio; MIMIC-IV: Medical Information Mart for Intensive Care-IV.

Subsequently, the subgroup analyses were stratified by sex (male and female), age (<65 and ≥65 years), BMI (<30 and ≥30 kg/m^2^), diabetes (yes and no), acute HF (yes and no), and insulin use (yes and no). No significant interactions were observed for sex, age, or acute HF subgroups (*P* for interaction>.05). Notably, significant interactions were detected between SHR and BMI (*P*=.04), diabetes (*P*=.01), and insulin use (*P*=.005; Figure 3), thus prompting further RCS analyses within these subgroups. Results indicated that in the subgroups with diabetes, insulin use, and BMI ≥30 kg/m^2^, a linear association was observed between SHR and sepsis incidence (all *P* overall<.05; *P* nonlinear>.05). In contrast, a U-shaped relationship was identified in the subgroup with BMI <30 kg/m^2^, with the inflection point at 1.03 (*P* overall=.01; *P* nonlinear=.09). No significant associations were found in the remaining subgroups (*P* overall>.05; Figures S7-S9 in Multimedia Appendix 1).

### Sensitivity Analyses

**Figure 4.**
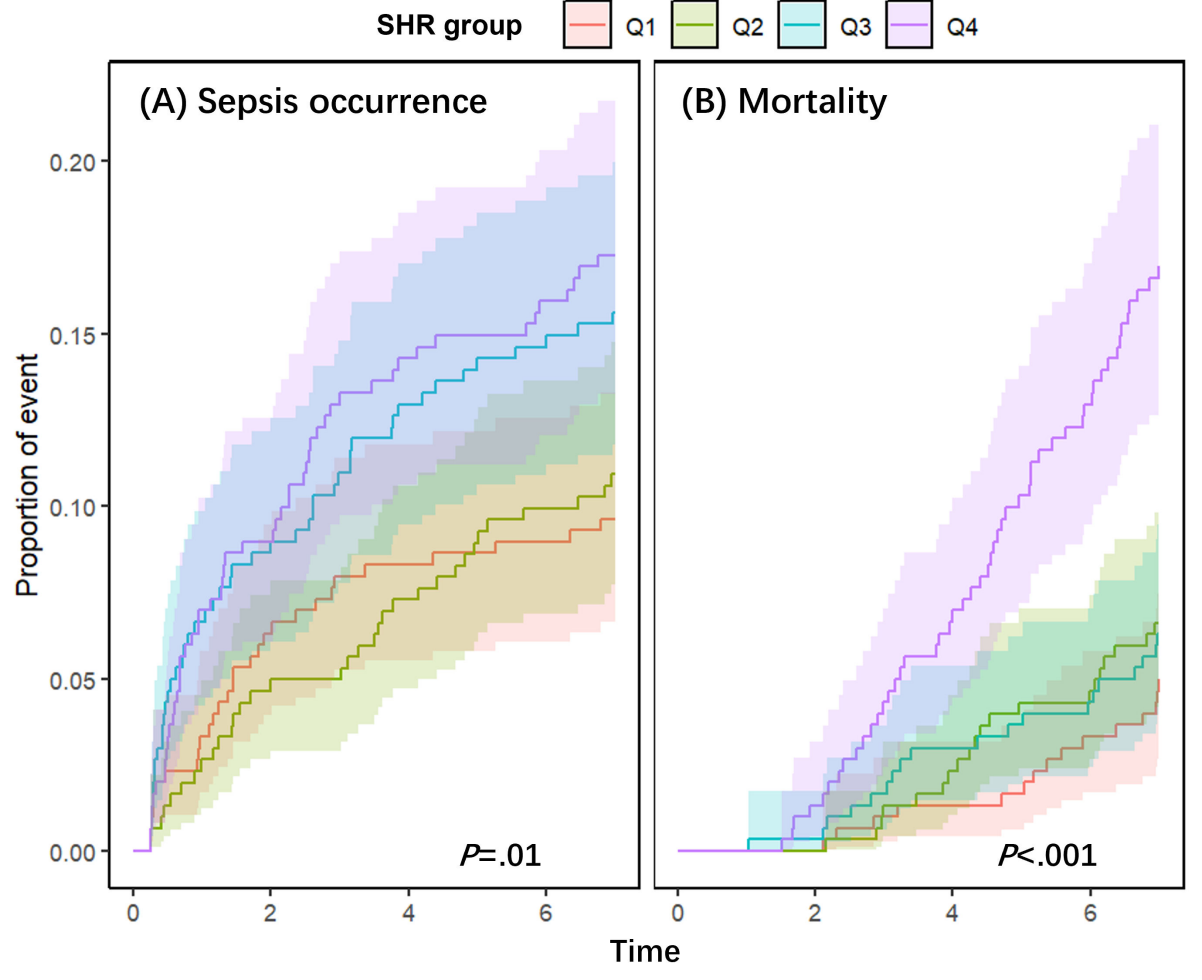
Cumulative incidence curves from Fine-Gray competing risk models, illustrating the impact of SHR quartiles on (A) the cumulative incidence of sepsis within 7 days and (B) the cumulative incidence of 7-day all-cause mortality, with each considered a competing event for the other. The analysis is based on a retrospective cohort of 1205 critically ill patients with heart failure from the MIMIC-IV database (Boston, MA, United States; 2008-2022). SHR quartiles: Q1 (<0.849); Q2 (0.849‐1.033); Q3 (1.033‐1.318); Q4 (>1.318). MIMIC-IV: Medical Information Mart for Intensive Care-IV; SHR: stress hyperglycemia ratio.

We performed different sensitivity tests to validate the robustness of our results. First, considering 7-day all-cause mortality as a competing event, the Fine-Gray competing risk model was used. In the univariable-adjusted Fine-Gray model, the cumulative occurrence of sepsis increased from Q1 to Q4 (*P*=.01), maintaining significance even when accounting for the competing risk of all-cause mortality (*P*<.001; Figure 4).

In the multivariable-adjusted Fine-Gray model, SHR, as a categorical variable (quartiles), revealed a significant overall difference in sepsis incidence across groups (*P*=.01). When modeled as a continuous variable in the fully adjusted model, the association was positive but not statistically significant (HR 1.18, 95% CI 0.96‐1.44; *P*=.11). There was a significant positive trend in sepsis risk across increasing SHR quartiles, indicating a dose-response relationship (*P* for trend<.001 in the fully adjusted model; Table S8 in Multimedia Appendix 1).

Second, the logistic regression was also made to show a significant positive association between SHR and sepsis risk (model 3: odds ratio 1.03, 95% CI 1.01‐1.05; *P*<.001), with sepsis risk increasing significantly across SHR quartiles (*P* for trend <.001; Table S9 in Multimedia Appendix 1).

Third, when the sepsis exclusion window was extended to 12, 24, 36, and 48 hours, the overall association remained consistent. However, the association was not significant when the exposure was modeled continuously in the fully adjusted model (all *P*>.05; Table S10 in Multimedia Appendix 1).

### Mediation Analysis

Table 3 shows the mediation value of inflammatory indices between SHR and sepsis occurrence.

**Table 3.** Mediation effects of inflammatory biomarkers on the stress hyperglycemia ratio-sepsis association^a^.

	Case, n	ACME^b^		ADE^c^		PE^d^		TE^e^	
		Estimate (95% CI)	*P* value	Estimate (95% CI)	*P* value	Estimate (95% CI)	*P* value	Estimate (95% CI)	*P* value
SII^f^	807	1.05 (1.01 to 1.13)	.01	1.90 (1.23 to 3.02)	.01	0.10 (0.01 to 0.30)	.01	2.00 (1.28 to 3.15)	.004
NLR^g^	807	1.03 (1.00 to 1.09)	.04	1.93 (1.23 to 2.97)	.004	0.06 (0.00 to 0.23)	.04	1.99 (1.29 to 3.04)	<.001
PLR^h^	807	1.02 (0.99 to 1.06)	.23	1.92 (1.26 to 2.93)	.004	0.04 (−0.02 to 0.14)	.23	1.96 (1.27 to 2.97)	<.001
MLR^i^	807	1.01 (0.97 to 1.06)	.53	1.97 (1.26 to 3.01)	<.001	0.03 (−0.06 to 0.13)	.53	1.99 (1.27 to 3.06)	<.001
NPR^j^	808	1.05 (1.00 to 1.12)	.04	1.88 (1.22 to 3.02)	.004	0.10 (0.00 to 0.30)	.04	1.97 (1.31 to 3.14)	.004
SIRI^k^	807	1.07 (1.00 to 1.15)	.04	1.78 (1.15 to 2.75)	.01	0.13 (0.00 to 0.39)	.05	1.90 (1.23 to 2.93)	.004
NMLR^l^	807	1.03 (1.00 to 1.09)	.04	1.93 (1.23 to 2.98)	.004	0.06 (0.00 to 0.23)	.04	1.99 (1.28 to 3.04)	<.001

a Mediation analysis estimating the ACMEs, ADEs, PM, and TEs of systemic inflammatory indices in the association between the stress hyperglycemia ratio and 7-day sepsis occurrence. Analysis was performed among 807 patients with complete inflammatory biomarker data from the Medical Information Mart for Intensive Care-IV database (Boston, MA, United States; 2008‐2022). Estimates were derived from a fully adjusted Cox regression model using 1000 bootstrap resamplings. The mediation effects presented in the table are estimated based on the fully adjusted model, which accounts for adjustments including sex, age, BMI, acute heart failure, diabetes, shock, Sequential Organ Failure Assessment score, antibiotic use, insulin use, and glucocorticoid use.

b ACME: average causal mediation effect (indirect effect).

c ADE: average direct effect.

d PE: proportion mediated.

e TE: total effect.

f SII: systemic immune-inflammation index.

g NLR: neutrophil-to-lymphocyte ratio.

h PLR: platelet-to-lymphocyte ratio.

i MLR: monocyte-to-lymphocyte ratio.

j NPR: neutrophil-to-platelet ratio.

k SIRI: systemic inflammation response index.

l NMLR: neutrophil-monocyte-to-lymphocyte ratio.

**Figure 5.**
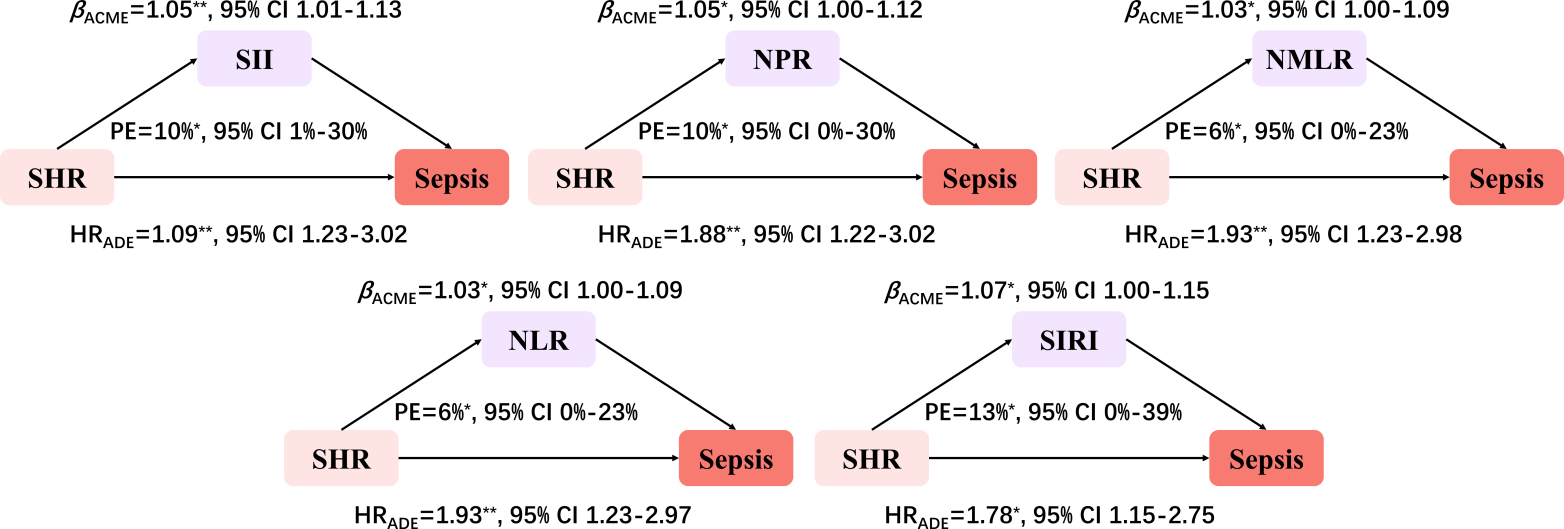
Mediation analysis (adjusted for sex, age, BMI, acute heart failure, diabetes, shock, Sequential Organ Failure Assessment score, antibiotic use, insulin use, and glucocorticoid use). ACME: average causal mediation effect; ADE: average direct effect; HR: hazard ratio; NLR: neutrophil-to-lymphocyte ratio; NMLR: neutrophil-monocyte-to-lymphocyte ratio; NPR: neutrophil-to-platelet ratio; PE: proportion mediated; SHR: stress hyperglycemia ratio; SII: systemic immune-inflammation index; SIRI: systemic inflammation response index. *P<.05; **P≤.01.

The mediation analysis indicates that, for the general population, the estimated proportions of the association mediated by NLR, NPR, NMLR, SIRI, and SII were 6% (95% CI 0.00%‐23%), 10% (95% CI 0.00%‐30%), 6% (95% CI 0.00%‐23%), 13% (95% CI 0.00%‐39%), and 10% (95% CI 1%‐30%), respectively (Figure 5).

For the diabetes subpopulation, no significant exposure mediator interaction effects were observed (all *P*>.05; Table S11 in Multimedia Appendix 1). Additionally, mediation analysis in the nondiabetic subgroup was underpowered due to limited sample size, resulting in unstable estimates with wide CIs (Table S12 in Multimedia Appendix 1).

## Discussion

### Principal Findings

In this study, we first demonstrated that a higher SHR was significantly associated with a higher risk of sepsis occurrence after fully adjusting for potential confounders in critically ill patients with HF based on the MIMIC-IV database, with an evidently positive linear relationship in the diabetes and BMI ≥30 kg/m^2^ subgroup. The association between SHR and sepsis occurrence in the RCS analysis after adjusting confounders further showed a nonlinear pattern, where risk increased up to an SHR threshold (1.29) and then plateaued. Furthermore, systemic inflammatory indices partially mediated this relationship, implicating inflammation as a potential mechanistic link.

### Comparison to Prior Work

The concept of SHR highlights a relative acute rise in glycemia compared to the individual’s previous glycemic status in response to a stress reaction or critical illness [11], which was triggered by the release of stress hormones (eg, adrenaline and cortisol), immune responses, neural activity, and insulin resistance [28].

Extensive research has shown that SHR was associated with adverse outcomes across various diseases. Consistent with our findings, elevated SHR has been linked to poorer prognosis in patients with acute coronary syndrome [12], acute HF [13], and sepsis [14,29,30]. However, while several studies have demonstrated a U-shaped association between SHR and mortality in various cardiovascular and metabolic diseases, including HF [13], acute myocardial infarction [31], and cardiovascular-kidney-metabolic syndrome [32], the relationship between SHR and the occurrence of sepsis in critically ill patients with HF remains underexplored. Our study revealed a positive, nonlinear saturation effect between SHR and sepsis occurrence in critically ill patients with HF. However, a U-shaped association between SHR and the occurrence of acute kidney injury in patients with congestive heart failure was observed in a study by Li et al [33], which contrasts with our finding of a plateauing effect. This discrepancy may be attributable to differences in the outcomes studied (sepsis vs acute kidney injury) and the competing risk of early mortality in patients with extreme SHR values, which was addressed in our sensitivity analyses using the Fine-Gray model.

The steep increase in risk below the SHR threshold of 1.29 in our study underscores a dose-dependent hazard, which likely reflects the cumulative, detrimental effects of acute hyperglycemia on immune dysfunction [8,9] and endothelial activation [34], making patients progressively more susceptible to infection. Several biological mechanisms may explain this association. First, critical illness activates the hypothalamic-pituitary-adrenal axis, leading to stress hyperglycemia [28], which impairs immune responses such as adhesion, chemotaxis, phagocytosis, and bacterial killing by immune cells [8], thus increasing susceptibility to infection. Second, stress hyperglycemia may cause endothelial dysfunction by increasing mitochondrial reactive oxygen species production [9], leading to upregulation of adhesion molecules and enhanced leukocyte recruitment [34], thereby amplifying the inflammatory cascade. Finally, hyperglycemia-driven reactive oxygen species generation can cause myocardial inflammation and dysfunction [35], worsening HF and indirectly elevating sepsis risk.

Our subgroup analyses further revealed that the association between SHR and sepsis was significantly modified by diabetes mellitus, BMI, and insulin use. RCS analysis showed a nonlinear saturation effect in the overall population, which was consistent in the diabetic subgroup. Specifically, in the diabetic subgroup, as SHR increased, the risk of sepsis increased linearly, a finding that aligns with previous studies, demonstrating that the long-term hyperglycemic state of patients with diabetes impairs immune cell function, including adhesion, chemotaxis, phagocytosis, and bacterial killing, thereby increasing susceptibility to sepsis [10,36]. The resulting chronic low-grade inflammation and vascular endothelial activation, typical in diabetes, also overlap with sepsis pathology [14,29], explaining why patients with HF and diabetes exhibited higher sepsis occurrence in our study.

While there is no definitive research to explain the risk plateau after SHR exceeds 1.29, we hypothesize the following potential factors. First, there is a significant competing risk of early mortality, given that patients with the highest SHR had the greatest 7-day mortality, potentially precluding the diagnosis of sepsis. Second, these patients likely receive more aggressive clinical management (eg, insulin therapy and broader-spectrum antibiotics), which may attenuate the observable sepsis risk. Therefore, an SHR >1.29 might be a stronger indicator of overall critical illness severity and metabolic derangement.

After adjusting for 7-day mortality as a competing event using the Fine-Gray model, SHR remained independently associated with an increased risk of sepsis. The subsequent sensitivity analysis further corroborated this finding, confirming the robustness of our results. Specifically, although the association was nonsignificant in the diabetes subgroup and nondiabetes subgroup when examined separately, the Q4 had the relatively highest 7-day all-cause mortality in the diabetes subgroup, while in the nondiabetes subgroup, patients in Q3 and Q4 groups had higher 7-day all-cause mortality than other groups.

Additionally, our study is the first to explore the mediating role of systemic inflammation in the SHR-sepsis association. We found that NLR, NPR, NMLR, SIRI, and SII significantly mediated this relationship, with mediation proportions ranging from 6% to 13%. This provides a new prospective between the proinflammatory effects of hyperglycemia [21,37-39] and the pathogenesis of sepsis [40], offering a mechanistic insight that has not been previously reported in this specific population. The potential mechanisms might be as follows: SHR underscores an individual’s acute glycemic response to stress, and the increased blood glucose further promotes monocyte and macrophage aggregation, leading to the release of additional proinflammatory cytokines [37-39], which contributes to inflammation, tissue damage, and impaired immune function.

Sepsis is a systemic inflammatory response syndrome caused by infection [3], wherein monocytes, neutrophils, and other innate immune cells release proinflammatory cytokines to induce infection [40]. Therefore, comprehensive inflammation indices might have certain mediation value for sepsis onset and prognosis, which is consistent with our findings. Chronic hyperglycemia in patients with diabetes may lead to adaptive changes in insulin signaling and glucose transporter expression, which can attenuate the acute inflammatory response [41]. Moreover, hospitalized individuals with diabetes typically receive stricter glucose management, potentially mitigating the detrimental impact of stress-induced hyperglycemia on sepsis risk and obscuring the role of inflammation mediation [42].

From a clinical perspective, these findings highlight that even moderate elevations in SHR (well below 1.29) might carry increased risk for sepsis, thus suggesting that it could serve as a dynamic and sensitive marker for early risk stratification. The identified threshold of 1.29 could serve as a practical tool for sepsis risk stratification, enabling earlier identification of high-risk patients for intensified monitoring and early treatment intervention based on this cutoff. From a public health perspective, SHR offers a low-cost, accessible biomarker for risk stratification, particularly in resource-limited settings. The finding that inflammatory indices mediate this relationship suggests that anti-inflammatory strategies, alongside strict glycemic control, might be beneficial in mitigating sepsis risk in this vulnerable population.

### Limitations

However, limitations exist. First, our retrospective cohort design, based on data from a public database, cannot establish a causal relationship between SHR and sepsis risk. Second, as HbA_1c_ is not routinely measured in all ICU admissions, restricting the cohort to patients with available HbA_1c_ may have introduced selection bias by overrepresenting individuals with known or suspected dysglycemia, thus limiting the generalizability of our findings to the broader population with HF. Third, the relatively small sample size of the diabetes subpopulation may contribute to the nonsignificant mediation effects observed in this subgroup. Fourth, the mediation analysis for the nondiabetic subgroup was underpowered due to the sample size, resulting in unstable estimates with wide CIs. Fifth, given the simultaneous measurement of SHR and inflammatory markers at baseline, the temporal sequence between exposure and mediators cannot be established, which was a significant constraint of the cross-sectional design. Sixth, the unavailability of data on inflammatory biomarkers, including C-reactive protein, procalcitonin, and interleukin-6, limited our ability to perform a comprehensive inflammation mediation analysis. Seventh, as antibiotic use is binary, the reported point-biserial correlation (*r*=−0.196) between SHR and antibiotic use is methodologically limited and should be interpreted descriptively. Finally, we were unable to account for HF phenotype (HF with reduced ejection fraction vs HF with preserved ejection fraction) due to data limitations.

### Future Directions

First, to translate our findings into clinical practice, future studies can commence with validating the prognostic utility of SHR in large, prospective studies. Second, researchers can delve into the underlying mechanisms connecting this metabolic ratio to sepsis susceptibility. Future studies should integrate SHR into a multivariate clinical prediction model to test whether it can effectively guide preventive strategies and improve outcomes in high-risk patients with HF.

### Conclusions

Elevated SHR was independently associated with increased 7-day sepsis risk in critically ill patients with HF, and this association was significantly modified by diabetes status. Furthermore, systemic inflammatory indices partially mediated the SHR and sepsis association in the overall cohort, suggesting inflammation as a potential mechanism. SHR may serve as a practical clinical marker for early identification of individuals at high risk of sepsis.

## Supplementary material

10.2196/81229 Multimedia Appendix 1 Supplementary tables and figures. This appendix contains all supplementary material for the retrospective cohort study of 1,205 critically ill patients with heart failure (MIMIC-IV database, 2008–2022), including: diagnostic codes for heart failure (Table S1); formulas for systemic inflammatory indices (Table S2); missing data and imputation methods (Table S3); baseline characteristics stratified by sepsis (Table S4) and diabetes status (Table S5); clinical outcomes by stress hyperglycemia ratio (SHR) quartiles (Table S6); interaction analysis (Table S7); Fine‑Gray competing risk models (Table S8); logistic regression sensitivity analysis (Table S9); competing risk analysis after excluding early sepsis diagnoses (Table S10); mediation analyses in diabetic (Table S11) and non‑diabetic (Table S12) subgroups; distribution of time‑to‑sepsis onset (Figure S1); scatter plots of SHR with antibiotic use (Figure S2) and SOFA score (Figure S3); Kaplan‑Meier curves for 7‑day mortality overall (Figure S4), in diabetic (Figure S5) and non‑diabetic (Figure S6) patients; and restricted cubic spline curves for SHR and sepsis risk stratified by diabetes (Figure S7), insulin use (Figure S8), and body mass index (Figure S9).

## Abbreviations

HbA_1c_: glycated hemoglobin
HF: heart failure
HIPAA: Health Insurance Portability and Accountability Act
HR: hazard ratio
ICD-10: International Classification of Diseases, Tenth Revision
ICD-9: International Classification of Diseases, Ninth Revision
ICU: intensive care unit
MIMIC-IV: Medical Information Mart for Intensive Care-IV
NLR: neutrophil-to-lymphocyte ratio
NMLR: neutrophil-monocyte-to-lymphocyte ratio
NPR: neutrophil-to-platelet ratio
RCS: restricted cubic splines
SHR: stress hyperglycemia ratio
SII: systemic immune-inflammation index
SIRI: systemic inflammation response index
SOFA: Sequential Organ Failure Assessment
STROBE: Strengthening the Reporting of Observational Studies in Epidemiology
