# Supplementary material for: Stress Hyperglycemia Ratio and the Risk of Sepsis in Patients With Heart Failure: Retrospective Cohort Study From Medical Information Mart for Intensive Care-IV
Source: JMIR Med Inform. 2026 Apr 17;14:e81229. doi: 10.2196/81229 (PMC13089799; doi:10.2196/81229)
Supplement: Multimedia Appendix 1 [file medinform-v14-e81229-s001.docx]

**Table S1**:  International Classification of Diseases, Ninth and Tenth Revision (ICD-9 and ICD-10) diagnostic codes used to define Heart Failure (HF) in a retrospective cohort study of critically ill patients admitted to the intensive care unit (ICU) at Beth Israel Deaconess Medical Center (Boston, MA, USA) from 2008 to 2022, utilizing data from the MIMIC-IV database

| Code System | Code |
| --- | --- |
| ICD-9 | 39891 |
|  | 40201 |
|  | 40211 |
|  | 40291 |
|  | 40401 |
|  | 40403 |
|  | 40411 |
|  | 40413 |
|  | 40491 |
|  | 40493 |
|  | 4280 |
|  | 4281 |
|  | 42820 |
|  | 42821 |
|  | 42822 |
|  | 42823 |
|  | 42830 |
|  | 42831 |
|  | 42832 |
|  | 42833 |
|  | 42840 |
|  | 42841 |
|  | 42842 |
|  | 42843 |
| ICD-10 | I0981 |
|  | I110 |
|  | I130 |
|  | I132 |
|  | I502 |
|  | I5020 |
|  | I5021 |
|  | I5022 |
|  | I5023 |
|  | I503 |
|  | I5030 |
|  | I5031 |
|  | I5032 |
|  | I5033 |
|  | I504 |
|  | I5040 |
|  | I5041 |
|  | I5042 |
|  | I5043 |
|  | I508 |
|  | I5081 |
|  | I50810 |
|  | I50811 |
|  | I50812 |
|  | I50813 |
|  | I50814 |
|  | I5082 |
|  | I5083 |
|  | I5084 |
|  | I5089 |

ICD 9: International Classification of Diseases 9; ICD 10: International Classification of Diseases 10.

# **Table S2.** Calculation formulas for systemic inflammatory indices (NLR, MLR, NMLR, NPR, PLR, SIRI, SII) used in the mediation analysis of the association between Stress Hyperglycemia Ratio and sepsis risk in a retrospective cohort study of 1,205 critically ill patients with heart failure admitted to the intensive care unit at Beth Israel Deaconess Medical Center (Boston, MA, USA) from 2008 to 2022, utilizing data from the MIMIC-IV database.

| Variable | Calculation formula |
| --- | --- |
| NLR | Neutrophil counts / Lymphocyte counts |
| MLR | Monocyte counts / Lymphocyte counts |
| NMLR | (Monocyte counts + Neutrophil counts) / Lymphocyte counts |
| NPR | Neutrophil counts / Platelet counts |
| PLR | Platelet counts / Lymphocyte counts |
| SIRI | Neutrophil counts × Monocyte counts / Lymphocyte counts |
| SII | Platelet counts × Neutrophil counts / Lymphocyte counts |

**Table S3.** List of baseline clinical variables, their missing data rates, and the imputation methods employed (Multiple Imputation by Chained Equations, MICE) in a retrospective cohort study of 1,205 critically ill patients with heart failure admitted to the intensive care unit at Beth Israel Deaconess Medical Center (Boston, MA, USA) from 2008 to 2022, utilizing data from the MIMIC-IV database.

| Variable | Missing rate | Methods |
| --- | --- | --- |
| CRP | 96.85% | Exclude |
| LD/LDH | 72.61% | Exclude |
| CK | 67.05% | Exclude |
| NT-proBNP | 63.98% | Exclude |
| Ck-MB | 57.68% | Exclude |
| LDL-C | 53.44% | Exclude |
| HDLC | 52.86% | Exclude |
| Triglycerides | 52.78% | Exclude |
| Bilirubin | 51.95% | Exclude |
| Cholesterol | 51.87% | Exclude |
| AST | 50.62% | Exclude |
| TNT | 50.62% | Exclude |
| ALT | 50.37% | Exclude |
| Neutrophils | 32.95% | Exclude |
| Basophils_ | 32.95% | Exclude |
| Eosinophils | 32.95% | Exclude |
| Lymphocytes | 32.95% | Exclude |
| Monocytes | 32.95% | Exclude |
| Intake | 22.32% | Mice |
| BMI | 20.41% | Mice |
| Temperature | 7.88% | Mice |
| PTT | 6.39% | Mice |
| PT | 6.14% | Mice |
| Sodium | 5.15% | Mice |
| Potassium | 5.06% | Mice |
| Sofa | 3.57% | Mice |
| Chloride | 1.49% | Mice |
| Bicarbonate | 1.49% | Mice |
| Creatinine | 1.00% | Mice |
| BUN | 0.83% | Mice |
| Platelets | 0.66% | Mice |
| Hemoglobin | 0.58% | Mice |
| WBC | 0.58% | Mice |
| Hematocrit | 0.50% | Mice |
| Heart rate | 0.25% | Mice |
| SBP | 0.25% | Mice |
| DBP | 0.25% | Mice |
| MBP | 0.25% | Mice |
| Respiratory rate | 0.25% | Mice |
| SHR | 0.00% | / |
| Gender | 0.00% | / |
| Age | 0.00% | / |
| Race | 0.00% | / |
| Acute HF | 0.00% | / |
| Hypertension | 0.00% | / |
| Coronary heart disease | 0.00% | / |
| AMI | 0.00% | / |
| Valve_disorder | 0.00% | / |
| AF | 0.00% | / |
| Chronic pulmonary disease | 0.00% | / |
| CKD | 0.00% | / |
| Liver disease | 0.00% | / |
| Cancer | 0.00% | / |
| Diabetes | 0.00% | / |
| Lipoprotein metabolism disorders | 0.00% | / |
| Shock | 0.00% | / |
| Charlson comorbidity | 0.00% | / |
| Apsiii | 0.00% | / |
| Sapsii | 0.00% | / |
| Sirs | 0.00% | / |
| Sofa | 0.00% | / |
| ACEI | 0.00% | / |
| Amiodarone | 0.00% | / |
| Anticoagulants_drug | 0.00% | / |
| Diuretic drug | 0.00% | / |
| NSAID | 0.00% | / |
| Beta blocker | 0.00% | / |
| Hypolipidemic drug | 0.00% | / |
| Antibiotic | 0.00% | / |
| Insulin | 0.00% | / |
| Glucocorticoid | 0.00% | / |
| Oral hypoglycemic drugs | 0.00% | / |

# CRP: C-Reactive Protein; LD/LDH: Lactate Dehydrogenase; CK: Creatine Kinase; NT-proBNP: N-terminal pro B-type Natriuretic Peptide; Ck-MB: Creatine Kinase-MB; LDL-C: Low-Density Lipoprotein Cholesterol; HDLC: High-Density Lipoprotein Cholesterol; AST: Aspartate Aminotransferase; ALT: Alanine Aminotransferase; TNT: Troponin T; BMI: Body Mass Index; PTT: Partial Thromboplastin Time; PT: Prothrombin Time; Sofa: Sequential Organ Failure Assessment; BUN: Blood Urea Nitrogen; WBC: White Blood Cell Count; SBP: Systolic Blood Pressure; DBP: Diastolic Blood Pressure; MBP: Mean Blood Pressure; SHR: Stress Hyperglycemia Ratio; HF: Heart Failure; AMI: Acute Myocardial Infarction; AF: Atrial Fibrillation; CKD: Chronic Kidney Disease; ACEI: Angiotensin-Converting Enzyme Inhibitor; NSAID: Non-Steroidal Anti-Inflammatory Drug; MICE: multiple imputation by chained equations.

# **Table S4.** Baseline demographic, clinical, laboratory, and treatment characteristics of 1,205 critically ill patients with heart failure in a retrospective cohort study, stratified by the occurrence of sepsis within 7 days of ICU admission. Patients were admitted to the intensive care unit at Beth Israel Deaconess Medical Center (Boston, MA, USA) from 2008 to 2022, utilizing data from the MIMIC-IV database.

|  | Overall (N=1205) | | No sepsis (N=1043) | | Sepsis (N=162) | P value |
| --- | --- | --- | --- | --- | --- | --- |
| Male, % | 764 (63.4) | | 667 (64.0) | | 97 (59.9) | 0.361 |
| Age, years | 71.51 [62.45, 79.47] | | 71.61 [62.33, 79.85] | | 70.95 [62.77, 78.56] | 0.339 |
| Race, % |  | |  | |  | 0.870 |
| White | 800 (66.4) | | 695 (66.6) | | 105 (64.8) |  |
| Black | 108 (9.0) | | 92 (8.8) | | 16 (9.9) |  |
| Others | 297 (24.6) | | 256 (24.5) | | 41 (25.3) |  |
| BMI, kg/m2 | 28.54 [24.42, 32.92] | | 28.40 [24.28, 32.91] | | 28.73 [25.19, 32.93] | 0.321 |
| HbA1c, % | 6.00 [5.60, 7.10] | | 6.10 [5.60, 7.15] | | 5.90 [5.50, 6.80] | 0.103 |
| Glucose, mmol/L | 133.00 [106.00, 182.00] | | 131.00 [106.00, 182.00] | | 140.50 [118.00, 184.00] | 0.009 |
| SHR | 1.03 [0.85, 1.32] | | 1.02 [0.84, 1.29] | | 1.13 [0.92, 1.46] | 0.001 |
| **Vital signs** |  | |  | |  |  |
| Heart rate, bpm | 81.28 [73.59, 91.48] | | 80.85 [73.30, 90.08] | | 86.74 [78.13, 96.50] | <0.001 |
| SBP, mmHg | 111.48 [104.13, 122.04] | | 111.77 [104.67, 122.20] | | 108.20 [100.48, 121.56] | 0.004 |
| DBP, mmHg | 60.85 [53.94, 69.63] | | 60.73 [53.95, 69.62] | | 62.03 [53.89, 69.91] | 0.835 |
| MBP, mmHg | 75.91 [70.45, 83.49] | | 75.81 [70.46, 83.28] | | 76.26 [70.49, 85.40] | 0.634 |
| Respiratory rate, br/min | 19.08 [17.22, 21.06] | | 19.00 [17.16, 20.98] | | 19.70 [18.00, 22.28] | 0.001 |
| Temperature, ℃ | 37.06 [36.83, 37.44] | | 37.06 [36.83, 37.40] | | 37.11 [36.84, 37.61] | 0.128 |
| Intake, ml/6h | 499.86 [227.77, 1221.86] | | 479.99 [228.47, 1132.91] | | 822.03 [220.91, 1874.50] | 0.003 |
| **Laboratory test** | |  | |  | |  |
| Hemoglobin, g/dL | 12.00 [10.40, 13.70] | | 12.00 [10.40, 13.70] | | 11.90 [10.33, 13.28] | 0.422 |
| White blood cell, 10^9^/L | 12.10 [8.80, 16.60] | | 11.80 [8.70, 16.40] | | 13.10 [9.55, 18.10] | 0.024 |
| Platelets, 10^9^/L | 217.00 [176.00, 274.00] | | 215.00 [175.00, 269.00] | | 233.50 [181.25, 295.50] | 0.007 |
| HCT, % | 36.80 [31.90, 41.20] | | 36.80 [31.90, 41.30] | | 36.45 [31.70, 40.30] | 0.410 |
| BUN, mg/dL | 24.00 [18.00, 36.00] | | 23.00 [17.00, 36.00] | | 26.00 [20.00, 39.75] | 0.012 |
| Creatinine, umol/L | 1.20 [0.90, 1.60] | | 1.20 [0.90, 1.60] | | 1.25 [1.00, 1.70] | 0.205 |
| Chloride, mmol/L | 104.00 [100.00, 107.00] | | 104.00 [100.00, 107.00] | | 102.00 [98.00, 105.00] | <0.001 |
| Sodium, mmol/L | 139.00 [136.00, 141.00] | | 139.00 [136.00, 141.00] | | 138.00 [135.00, 141.00] | 0.023 |
| Potassium, mmol/L | 4.40 [4.00, 4.80] | | 4.40 [4.00, 4.80] | | 4.40 [4.10, 4.80] | 0.155 |
| Bicarbonate, mmol/L | 25.00 [22.00, 27.00] | | 25.00 [22.00, 27.00] | | 24.00 [21.00, 27.00] | 0.251 |
| PT, s | 14.70 [12.90, 17.40] | | 14.70 [12.90, 17.30] | | 14.80 [13.10, 18.00] | 0.403 |
| APTT, s | 41.50 [30.90, 73.80] | | 40.70 [30.90, 72.25] | | 48.60 [30.22, 93.42] | 0.114 |
| **Comorbidities** | |  | |  | |  |
| Acute HF，% | 855 (71.0) | | 719 (68.9) | | 136 (84.0) | <0.001 |
| Hypertension, % | 297 (24.6) | | 257 (24.6) | | 40 (24.7) | 1.000 |
| Coronary heart disease, % | 934 (77.5) | | 810 (77.7) | | 124 (76.5) | 0.829 |
| AMI, % | 551 (45.7) | | 476 (45.6) | | 75 (46.3) | 0.943 |
| Valve disorder, % | 540 (44.8) | | 455 (43.6) | | 85 (52.5) | 0.043 |
| Atrial fibrillation, % | 604 (50.1) | | 505 (48.4) | | 99 (61.1) | 0.003 |
| COPD, % | 329 (27.3) | | 282 (27.0) | | 47 (29.0) | 0.667 |
| Chronic kidney disease, % | 421 (34.9) | | 361 (34.6) | | 60 (37.0) | 0.607 |
| Liver disease, % | 74 (6.1) | | 63 (6.0) | | 11 (6.8) | 0.846 |
| Cancer, % | 40 (3.3) | | 32 (3.1) | | 8 (4.9) | 0.317 |
| Diabetes, % | 564 (46.8) | | 492 (47.2) | | 72 (44.4) | 0.574 |
| Lipoprotein metabolism disorders, % | 800 (66.4) | | 697 (66.8) | | 103 (63.6) | 0.469 |
| Shock, % | 284 (23.6) | | 205 (19.7) | | 79 (48.8) | <0.001 |
| pneumonia (%) | 104 (8.6) | | 61 (5.8) | | 43 (26.5) | <0.001 |
| **Disease severity score** | |  | |  | |  |
| Charlson comorbidity index | 6.00 [4.00, 8.00] | | 6.00 [4.00, 8.00] | | 7.00 [4.00, 8.00] | 0.220 |
| APSIII | 40.00 [31.00, 50.00] | | 39.00 [31.00, 49.00] | | 47.00 [35.00, 59.75] | <0.001 |
| SAPSII | 35.00 [28.00, 43.00] | | 35.00 [28.00, 42.00] | | 39.00 [30.25, 48.75] | 0.001 |
| SIRS | 3.00 [2.00, 3.00] | | 3.00 [2.00, 3.00] | | 3.00 [2.00, 3.00] | <0.001 |
| SOFA | 3.00 [1.00, 5.00] | | 3.00 [1.00, 5.00] | | 2.00 [1.00, 4.00] | 0.059 |
| **Medication** | |  | |  | |  |
| ACEI/ARB, % | 398 (33.0) | | 351 (33.7) | | 47 (29.0) | 0.281 |
| Amiodarone, % | 154 (12.8) | | 123 (11.8) | | 31 (19.1) | 0.013 |
| Anticoagulants, % | 139 (11.5) | | 120 (11.5) | | 19 (11.7) | 1.000 |
| NSAID, % | 770 (63.9) | | 665 (63.8) | | 105 (64.8) | 0.863 |
| Beta blocker, % | 737 (61.2) | | 642 (61.6) | | 95 (58.6) | 0.535 |
| Diuretic drug, % | 751 (62.3) | | 638 (61.2) | | 113 (69.8) | 0.044 |
| Hypolipidemic drug, % | 751 (62.3) | | 650 (62.3) | | 101 (62.3) | 1.000 |
| Antibiotics, % | 595 (49.4) | | 505 (48.4) | | 90 (55.6) | 0.108 |
| Insulin, % | 747 (62.0) | | 666 (63.9) | | 81 (50.0) | 0.001 |
| Glucocorticoid, % | 167 (13.9) | | 141 (13.5) | | 26 (16.0) | 0.456 |
| Oral hypoglycemic drugs, % | 19 (1.6) | | 18 (1.7) | | 1 (0.6) | 0.475 |

SHR: stress hyperglycemia ratio; BMI: Body Mass Index; HbA1c: Glycated Hemoglobin; SBP: Systolic Blood Pressure; DBP: Diastolic Blood Pressure; MBP: Mean Blood Pressure; HCT: Hematocrit; BUN: Blood Urea Nitrogen; PT: Prothrombin Time; APTT: Activated Partial Thromboplastin Time; HF: Heart Failure; AMI: Acute Myocardial Infarction; COPD: Chronic Obstructive Pulmonary Disease; APSIII: Acute Physiology Score III; SAPSII: Simplified Acute Physiology Score II; SIRS: Systemic Inflammatory Response Syndrome; SOFA: Sequential Organ Failure Assessment; ACEI/ARB: Angiotensin-Converting Enzyme Inhibitor/Angiotensin Receptor Blocker; NSAID: Non-steroidal Anti-inflammatory Drugs.

# **Table S5.** Baseline characteristics of 1,205 critically ill patients with heart failure in a retrospective cohort study, stratified by diabetes status. Patients were admitted to the intensive care unit at Beth Israel Deaconess Medical Center (Boston, MA, USA) from 2008 to 2022, utilizing data from the MIMIC-IV database.

|  | Overall (N=1205) | | No diabetes (N=641) | | Diabetes (N=564) | P value |
| --- | --- | --- | --- | --- | --- | --- |
| Male, % | 764 (63.4) | | 417 (65.1) | | 347 (61.5) | 0.227 |
| Age, years | 71.51 [62.45, 79.47] | | 71.69 [61.80, 80.43] | | 71.37 [63.18, 78.78] | 0.714 |
| Race, % |  | |  | |  | 0.095 |
| White | 800 (66.4) | | 440 (68.6) | | 360 (63.8) |  |
| Black | 108 (9.0) | | 48 (7.5) | | 60 (10.6) |  |
| Others | 297 (24.6) | | 153 (23.9) | | 144 (25.5) |  |
| BMI, kg/m2 | 28.54 [24.42, 32.92] | | 27.90 [23.79, 31.94] | | 28.96 [25.30, 34.15] | <0.001 |
| HbA1c, % | 6.00 [5.60, 7.10] | | 5.70 [5.40, 6.00] | | 7.10 [6.30, 8.30] | <0.001 |
| Glucose, mmol/L | 133.00 [106.00, 182.00] | | 117.00 [101.00, 147.00] | | 163.00 [121.75, 226.00] | <0.001 |
| SHR | 1.03 [0.85, 1.32] | | 1.02 [0.88, 1.24] | | 1.06 [0.80, 1.37] | 0.785 |
| **Vital signs** |  | |  | |  |  |
| Heart rate, bpm | 81.28 [73.59, 91.48] | | 81.23 [73.80, 91.89] | | 81.47 [73.38, 90.64] | 0.625 |
| SBP, mmHg | 111.48 [104.13, 122.04] | | 110.12 [102.68, 119.83] | | 112.76 [105.14, 124.14] | <0.001 |
| DBP, mmHg | 60.85 [53.94, 69.63] | | 62.30 [54.70, 70.73] | | 59.36 [53.12, 67.39] | <0.001 |
| MBP, mmHg | 75.91 [70.45, 83.49] | | 76.48 [70.70, 84.54] | | 75.18 [70.30, 82.59] | 0.024 |
| Respiratory rate, br/min | 19.08 [17.22, 21.06] | | 19.38 [17.48, 21.50] | | 18.80 [16.93, 20.59] | <0.001 |
| Temperature, ℃ | 37.06 [36.83, 37.44] | | 37.06 [36.83, 37.44] | | 37.06 [36.89, 37.50] | 0.759 |
| Intake, ml/6h | 499.86 [227.77, 1221.86] | | 506.26 [224.73, 1169.06] | | 494.58 [229.34, 1264.91] | 0.945 |
| **Laboratory test** | |  | |  | |  |
| Hemoglobin, g/dL | 12.00 [10.40, 13.70] | | 12.50 [10.80, 14.00] | | 11.40 [10.00, 13.10] | <0.001 |
| Write blood cell, 10^9^/L | 12.10 [8.80, 16.60] | | 12.20 [8.80, 17.10] | | 11.80 [8.80, 16.12] | 0.204 |
| Platelets, 10^9^/L | 217.00 [176.00, 274.00] | | 215.00 [176.00, 271.00] | | 221.00 [175.75, 275.00] | 0.563 |
| HCT, % | 36.80 [31.90, 41.20] | | 38.10 [32.90, 42.50] | | 35.10 [30.87, 39.60] | <0.001 |
| BUN, mg/dL | 24.00 [18.00, 36.00] | | 22.00 [17.00, 32.00] | | 26.00 [19.00, 42.25] | <0.001 |
| Creatinine, umol/L | 1.20 [0.90, 1.60] | | 1.10 [0.90, 1.50] | | 1.30 [1.00, 1.70] | <0.001 |
| Chloride, mmol/L | 104.00 [100.00, 107.00] | | 104.00 [100.00, 107.00] | | 103.00 [99.00, 106.00] | 0.016 |
| Sodium, mmol/L | 139.00 [136.00, 141.00] | | 139.00 [137.00, 141.00] | | 138.00 [136.00, 141.00] | 0.005 |
| Potassium, mmol/L | 4.40 [4.00, 4.80] | | 4.30 [4.00, 4.70] | | 4.40 [4.10, 4.90] | 0.004 |
| Bicarbonate, mmol/L | 25.00 [22.00, 27.00] | | 25.00 [23.00, 27.00] | | 24.00 [22.00, 27.00] | 0.239 |
| PT, s | 14.70 [12.90, 17.40] | | 14.70 [12.90, 17.30] | | 14.70 [13.00, 17.50] | 0.700 |
| APTT, s | 41.50 [30.90, 73.80] | | 40.30 [30.00, 77.50] | | 42.80 [31.30, 70.80] | 0.770 |
| **Comorbidities** | |  | |  | |  |
| Acute HF，% | 855 (71.0) | | 457 (71.3) | | 398 (70.6) | 0.831 |
| Hypertension, % | 297 (24.6) | | 172 (26.8) | | 125 (22.2) | 0.070 |
| Coronary heart disease, % | 934 (77.5) | | 444 (69.3) | | 490 (86.9) | <0.001 |
| AMI, % | 551 (45.7) | | 302 (47.1) | | 249 (44.1) | 0.331 |
| Valve disorder, % | 551 (45.7) | | 302 (47.1) | | 249 (44.1) | 0.331 |
| Atrial fibrillation, % | 604 (50.1) | | 343 (53.5) | | 261 (46.3) | 0.014 |
| COPD, % | 329 (27.3) | | 175 (27.3) | | 154 (27.3) | 1.000 |
| Chronic kidney disease, % | 421 (34.9) | | 173 (27.0) | | 248 (44.0) | <0.001 |
| Liver disease, % | 74 (6.1) | | 39 (6.1) | | 35 (6.2) | 1.000 |
| Cancer, % | 40 (3.3) | | 19 (3.0) | | 21 (3.7) | 0.567 |
| Lipoprotein metabolism disorders, % | 800 (66.4) | | 367 (57.3) | | 433 (76.8) | <0.001 |
| Shock, % | 284 (23.6) | | 159 (24.8) | | 125 (22.2) | 0.312 |
| pneumonia (%) | 104 (8.6) | | 58 (9.0) | | 46 (8.2) | 0.654 |
| **Disease severity score** | |  | |  | |  |
| Charlson comorbidity index | 6.00 [4.00, 8.00] | | 5.00 [4.00, 7.00] | | 7.00 [6.00, 9.00] | <0.001 |
| Apsiii | 40.00 [31.00, 50.00] | | 38.00 [30.00, 49.00] | | 42.00 [33.00, 51.00] | <0.001 |
| Sapsii | 35.00 [28.00, 43.00] | | 34.00 [27.00, 43.00] | | 35.00 [29.00, 43.00] | 0.064 |
| Sirs | 3.00 [2.00, 3.00] | | 3.00 [2.00, 3.00] | | 3.00 [2.00, 3.00] | 0.950 |
| Sofa | 3.00 [1.00, 5.00] | | 3.00 [1.00, 5.00] | | 3.00 [1.00, 5.00] | 0.035 |
| **Medication at baseline** | |  | |  | |  |
| ACEI/ARB, % | 398 (33.0) | | 202 (31.5) | | 196 (34.8) | 0.258 |
| Amiodarone, % | 154 (12.8) | | 104 (16.2) | | 50 (8.9) | <0.001 |
| Anticoagulants, % | 139 (11.5) | | 80 (12.5) | | 59 (10.5) | 0.315 |
| NSAID, % | 770 (63.9) | | 382 (59.6) | | 388 (68.8) | 0.001 |
| Beta blocker, % | 737 (61.2) | | 363 (56.6) | | 374 (66.3) | 0.001 |
| Diuretic drug, % | 751 (62.3) | | 389 (60.7) | | 362 (64.2) | 0.234 |
| Hypolipidemic drug, % | 751 (62.3) | | 361 (56.3) | | 390 (69.1) | <0.001 |
| Antibiotics, % | 595 (49.4) | | 293 (45.7) | | 302 (53.5) | 0.008 |
| Insulin, % | 747 (62.0) | | 254 (39.6) | | 493 (87.4) | <0.001 |
| Glucocorticoid, % | 167 (13.9) | | 81 (12.6) | | 86 (15.2) | 0.220 |
| Oral hypoglycemic drugs, % | 19 (1.6) | | 0 (0.0) | | 19 (3.4) | <0.001 |

Specific definitions refer to Table 1

# **Table S6.** Clinical outcomes (7-day sepsis, 7-day mortality, 7-day composite outcome of sepsis or death, 28-day mortality, and in-hospital mortality) stratified by quartiles of the Stress Hyperglycemia Ratio among 1,205 critically ill patients with heart failure admitted to the intensive care unit at Beth Israel Deaconess Medical Center (Boston, MA, USA) from 2008 to 2022, utilizing data from the MIMIC-IV database.

|  | Overall | Groups of SHR quartile ^a^ | | | | P value |
| --- | --- | --- | --- | --- | --- | --- |
|  |  | Q1 (N=301) | Q2 (N=302) | Q3 (N=301) | Q4 (N=301) |  |
| Sepsis within 7day | 162 (13.4) | 29 (9.6) | 34 (11.3) | 47 (15.6) | 52 (17.3) | 0.018 |
| Mortality within 7day | 110 (9.1) | 18 (6.0) | 21 (7.0) | 19 (6.3) | 52 (17.3) | <0.001 |
| Composite outcome ^b^ within 7day | 266 (22.1) | 44 (14.6) | 53 (17.5) | 66 (21.9) | 103 (34.2) | <0.001 |
| 28-days mortality | 343 (28.5) | 90 (29.9) | 78 (25.8) | 69 (22.9) | 106 (35.2) | 0.006 |
| Hospital mortality | 65 (5.4) | 15 (5.0) | 11 (3.6) | 14 (4.7) | 25 (8.3) | 0.065 |

^a^ SHR quartile: Q1: <0.8490; Q2: 0.8490-1.0331; Q3: 1.0331-1.3177; Q4: >1.3177.

SHR: stress hyperglycemia ratio.

^b^ Composite outcome within 7day: sepsis or died within 7days

**Table S7.** Interaction analysis between the Stress Hyperglycemia Ratio and key clinical factors (diabetes, BMI, insulin use) for 7-day sepsis risk among 1,205 critically ill patients with heart failure admitted to the intensive care unit at Beth Israel Deaconess Medical Center (Boston, MA, USA) from 2008 to 2022, utilizing data from the MIMIC-IV database. Hazard ratios for multiplicative interaction terms were estimated using multivariable Cox proportional hazards regression models adjusted for gender, age, BMI, acute heart failure, shock, SOFA score, antibiotic use, and glucocorticoid use.

|  | HR (95%CI) | P value |
| --- | --- | --- |
| SHR*DM | 1.96 (1.23-3.14) | 0.005 |
| SHR*BMI | 1.02 (0.99-1.05) | 0.166 |
| SHR*BMI_group | 1.56 (0.97-2.50) | 0.068 |
| SHR*Use_of_insulin | 0.48 (0.30-0.77) | 0.002 |

Hazard ratios and 95% confidence intervals for the multiplicative interaction terms were estimated using multivariable Cox proportional hazards regression models, adjusted for gender, age, body mass index, acute heart failure, shock, Sequential Organ Failure Assessment score, antibiotic use, and glucocorticoid use. SHR*DM: Hazard ratio for the multiplicative interaction term between SHR and diabetes mellitus. SHR*BMI: Interaction with BMI as a continuous variable. SHR*BMI_group: Interaction with BMI dichotomized at 30 kg/m². SHR*Use_of_insulin: Interaction with baseline insulin therapy. HR >1 indicates the association between SHR and sepsis is stronger in the specified subgroup. CI: confidence interval.

# **Table S8.** Fine-Gray subdistribution hazard models assessing the association between the Stress Hyperglycemia Ratio and 7-day sepsis occurrence, accounting for the competing risk of death, among 1,205 critically ill patients with heart failure admitted to the intensive care unit at Beth Israel Deaconess Medical Center (Boston, MA, USA) from 2008 to 2022, utilizing data from the MIMIC-IV database.

|  | Events / | Model 1 |  | Model 2 |  | Model 3 |  |
| --- | --- | --- | --- | --- | --- | --- | --- |
|  |  | HR (95% CI) | P value | HR (95% CI) | P value | HR (95% CI) | P value |
| SHR | / | 1.31 (1.08-1.59) | 0.006 | 1.34 (1.10-1.63) | 0.003 | 1.18 (0.96-1.44) | 0.110 |
| Q1 | 44/301 | 0.54 (0.34-0.85) | 0.007 | 0.54 (0.34-0.85) | 0.008 | 0.51 (0.32-0.82) | 0.005 |
| Q2 | 53 /301 | 0.60 (0.39-0.93) | 0.022 | 0.60 (0.39-0.93) | 0.022 | 0.60 (0.39-0.93) | 0.023 |
| Q3 | 66 /301 | 0.90 (0.60-1.33) | 0.590 | 0.90 (0.60-1.34) | 0.590 | 0.96 (0.65-1.42) | 0.840 |
| Q4 | 103 /301 | Reference |  | Reference |  | Reference |  |
| P for trends | / | 1.25 (1.09-1.44) | 0.002 | 1.25 (1.09-1.44) | 0.002 | 1.27 (1.10-1.46) | <0.001 |

Model 1: unadjusted model;

Model 2: adjusted by gender, age, BMI;

Model 3: Model2 plus acute HF, diabetes, shock, SOFA score, antibiotic, insulin, glucocorticoid;

SHR: stress hyperglycemia ratio; HF: heart failure

# **Table S9.** Sensitivity analysis using multivariable logistic regression models to assess the association between the Stress Hyperglycemia Ratio and the occurrence of sepsis within 7 days of ICU admission among 1,205 critically ill patients with heart failure admitted to the intensive care unit at Beth Israel Deaconess Medical Center (Boston, MA, USA) from 2008 to 2022, utilizing data from the MIMIC-IV database.

|  | Events / | Model 1 |  | Model 2 |  | Model 3 |  |
| --- | --- | --- | --- | --- | --- | --- | --- |
|  |  | OR (95% CI) | P value | OR (95% CI) | P value | OR (95% CI) | P value |
| SHR | / | 1.06 (1.02-1.10) | 0.002 | 1.06 (1.02-1.10) | 0.002 | 1.03 (1.01-1.05） | <0.001 |
| Q1 | 29/301 | 0.93 (0.88-0.98) | 0.006 | 0.93 (0.88-0.98) | 0.006 | 0.92 (0.87-0.97) | 0.002 |
| Q2 | 34 /301 | 0.94 (0.89-0.99) | 0.030 | 0.94 (0.89-0.99) | 0.032 | 0.94 (0.89-0.99) | 0.027 |
| Q3 | 47 /301 | 0.98 (0.93-1.04) | 0.549 | 0.98 (0.93-1.04) | 0.546 | 0.99 (0.94-1.04) | 0.681 |
| Q4 | 52 /301 | Reference |  | Reference |  | Reference |  |
| P for trends | / | 1.03 (1.01-1.05) | 0.002 | 1.03 (1.01-1.05) | 0.002 | 1.03 (1.01-1.05) | <0.001 |

Model 1: unadjusted model;

Model 2: adjusted by gender, age, BMI;

Model 3: Model2 plus acute HF, diabetes, shock, SOFA score, antibiotic, insulin, glucocorticoid;

SHR: stress hyperglycemia ratio; HF: heart failure.

# **Table S10.** Sensitivity analysis of the association between the Stress Hyperglycemia Ratio and 7-day sepsis risk using Fine-Gray competing risk models, after sequentially excluding patients diagnosed with sepsis within 12, 24, 36, and 48 hours of ICU admission. Analysis was conducted among 1,205 critically ill patients with heart failure admitted to the intensive care unit at Beth Israel Deaconess Medical Center (Boston, MA, USA) from 2008 to 2022, utilizing data from the MIMIC-IV database.

|  | Events / | Model 1 | | Model 2 | | Model 3 | |
| --- | --- | --- | --- | --- | --- | --- | --- |
|  |  | HR (95% CI) | P value | HR (95% CI) | P value | HR (95% CI) | P value |
| **Excluded those diagnosed sepsis within 12 hours** | | | | | | | |
| SHR | / | 1.29 (1.07-1.56) | 0.008 | 1.33 (1.09-1.63) | 0.005 | 1.15 (0.96-1.37) | 0.133 |
| Q1 | 22/294 | 0.50 (0.30-0.83) | 0.008 | 0.51 (0.30-0.85) | 0.010 | 0.48 (0.28-0.81) | 0.006 |
| Q2 | 30/298 | 0.67 (0.42-1.07) | 0.094 | 0.67 (0.42-1.08) | 0.098 | 0.66 (0.41-1.06) | 0.087 |
| Q3 | 34/288 | 0.80 (0.51-1.26) | 0.338 | 0.80 (0.51-1.26) | 0.338 | 0.66 (0.41-1.06) | 0.087 |
| Q4 | 42/291 | Reference |  | Reference |  | Reference |  |
| P for trends | / | 1.25 (1.07-1.46) | 0.005 | 1.25 (1.06-1.46) | 0.007 | 1.27 (1.08-1.50) | 0.004 |
| **Excluded those diagnosed sepsis within 24 hours** | | | | | | | |
| SHR | / | 1.25 (0.98-1.58) | 0.067 | 1.30 (1.01-1.67) | 0.043 | 1.13 (0.92-1.40) | 0.245 |
| Q1 | 19/291 | 0.58 (0.33-1.03) | 0.061 | 0.59 (0.33-1.04) | 0.068 | 0.53 (0.30-0.96) | 0.035 |
| Q2 | 26/294 | 0.78 (0.46-1.32) | 0.356 | 0.78 (0.46-1.32) | 0.354 | 0.71 (0.41-1.22) | 0.212 |
| Q3 | 27/281 | 0.86 (0.51-1.44) | 0.565 | 0.85 (0.51-1.43) | 0.549 | 0.91 (0.54-1.53) | 0.718 |
| Q4 | 31/280 | Reference |  | Reference |  | Reference |  |
| P for trends | / | 1.18 (0.99-1.41) | 0.060 | 1.18 (0.99-1.40) | 0.067 | 1.23 (1.03-1.47) | 0.025 |
| **Excluded those diagnosed sepsis within 36 hours** | | | | | | | |
| SHR | / | 1.27 (0.99-1.63) | 0.062 | 1.33 (1.02-1.73) | 0.036 | 1.14 (0.91-1.42) | 0.263 |
| Q1 | 13/285 | 0.47 (0.24-0.92) | 0.026 | 0.47 (0.24-0.92) | 0.027 | 0.43 (0.22-0.84) | 0.014 |
| Q2 | 22/290 | 0.79 (0.45-1.39) | 0.407 | 0.78 (0.44-1.38) | 0.398 | 0.72 (0.40-1.29) | 0.266 |
| Q3 | 22/276 | 0.83 (0.47-1.47) | 0.528 | 0.83 (0.47-1.46) | 0.516 | 0.89 (0.50-1.58) | 0.699 |
| Q4 | 26/275 | Reference |  | Reference |  | Reference |  |
| P for trends | / | 1.24 (1.02-1.51) | 0.031 | 1.24 (1.02-1.51) | 0.032 | 1.29 (1.06-1.58) | 0.013 |
| **Excluded those diagnosed sepsis within 48 hours** | | | | | | | |
| SHR | / | 1.33 (1.07-1.67) | 0.012 | 1.40 (1.11-1.77) | 0.005 | 1.17 (0.95-1.45) | 0.139 |
| Q1 | 10/282 | 0.38 (0.18-0.78) | 0.009 | 0.37 (0.18-0.77) | 0.008 | 0.34 (0.16-0.72) | 0.005 |
| Q2 | 19/287 | 0.70 (0.39-1.28) | 0.249 | 0.70 (0.39-1.27) | 0.243 | 0.64 (0.34-1.18) | 0.149 |
| Q3 | 20/274 | 0.79 (0.44-1.42) | 0.423 | 0.79 (0.44-1.42) | 0.424 | 0.85 (0.47-1.53) | 0.585 |
| Q4 | 25/274 | Reference |  | Reference |  | Reference |  |
| P for trends | / | 1.32 (1.07-1.63) | 0.009 | 1.33 (1.07-1.64) | 0.008 | 1.38 (1.11-1.71) | 0.003 |

Model 1: unadjusted model;

Model 2: adjusted by gender, age, BMI;

Model 3: Model2 plus acute HF, diabetes, shock, SOFA score, antibiotic, insulin, glucocorticoid;

SHR: stress hyperglycemia ratio; HF: heart failure

# **Table S11.** Mediation analysis examining the role of systemic inflammatory indices (SII, NLR, PLR, MLR, NPR, SIRI, NMLR) in the association between the Stress Hyperglycemia Ratio and 7-day sepsis occurrence, restricted to the diabetic subgroup (n = 564) of heart failure patients admitted to the intensive care unit at Beth Israel Deaconess Medical Center (Boston, MA, USA) from 2008 to 2022, utilizing data from the MIMIC-IV database. Estimates are based on a fully adjusted Cox regression model with 1,000 bootstrap resamplings.

|  | Case | ACME | | ADE | | PE | | TE | |
| --- | --- | --- | --- | --- | --- | --- | --- | --- | --- |
|  |  | Estimate (95%CI) | P value | Estimate (95%CI) | P value | Estimate (95%CI) | P value | Estimate (96%CI) | P value |
| SII | 363 | 1.04(0.95-1.16) | 0.342 | 2.21(1.32-4.15) | <0.001 | 0.07(-0.10-0.27) | 0.342 | 2.30 (1.36 - 4.39) | <0.001 |
| NLR | 363 | 1.02(0.93-1.13) | 0.574 | 2.22(1.29-4.15) | <0.001 | 0.03(-0.14-0.23) | 0.574 | 2.26 (1.34 - 4.36) | <0.001 |
| PLR | 363 | 1.00(0.90-1.06) | 0.986 | 2.22(1.34-4.33) | <0.001 | 0.01(-0.23-0.12) | 0.986 | 2.23 (1.35 - 4.11) | <0.001 |
| MLR | 363 | 1.00(0.93-1.06) | 0.948 | 2.28(1.38-4.44) | <0.001 | -0.01 (-0.14- 0.10) | 0.948 | 2.28 (1.34 - 4.41) | <0.001 |
| NPR | 364 | 1.01(0.96-1.09) | 0.718 | 2.35(1.39-4.60) | 0.006 | 0.02(-0.07-0.14) | 0.72 | 2.38 (1.43 - 4.61) | 0.006 |
| SIRI | 363 | 1.03(0.94-1.12) | 0.630 | 2.27(1.35-4.45) | <0.001 | 0.05(-0.12-0.21) | 0.63 | 2.33 (1.37 - 4.53) | <0.001 |
| NMLR | 363 | 1.02(0.93-1.13) | 0.592 | 2.22(1.30-4.16) | <0.001 | 0.04(-0.14-0.22) | 0.592 | 2.26 (1.34 - 4.38) | <0.001 |

ACME, average causal mediation effects (indirect effect); ADE, average direct effects; PE, proportion mediated; TE: total effect；SII, systemic immune - inflammation index; NLR, neutrophil - to - lymphocyte ratio; PLR, platelet - to - lymphocyte ratio; MLR, monocyte - to - lymphocyte ratio; NPR, neutrophil - to - platelet ratio; SIRI, systemic inflammation response index; NMLR, neutrophil - monocyte - to - lymphocyte ratio. The mediation effects presented in the table are estimated based on the fully adjusted model, which accounts for adjustments including gender, age, BMI, acute heart failure, diabetes, shock, SOFA score, antibiotic, insulin, glucocorticoid.

**Table S12.^[[1]](#footnote-1)^** Mediation analysis examining the role of systemic inflammatory indices (SII, NLR, PLR, MLR, NPR, SIRI, NMLR) in the association between the Stress Hyperglycemia Ratio and 7-day sepsis occurrence, restricted to the non-diabetic subgroup (n = 641) of heart failure patients admitted to the intensive care unit at Beth Israel Deaconess Medical Center (Boston, MA, USA) from 2008 to 2022, utilizing data from the MIMIC-IV database. Estimates are based on a fully adjusted Cox regression model with 1,000 bootstrap resamplings. Footnote: Estimates for the non-diabetic subgroup should be interpreted with caution due to the limited sample size and wide confidence intervals, which indicate statistical instability and reduced power for mediation analysis in this subgroup.

|  | Case | ACME | | ADE | | PE | | TE | |
| --- | --- | --- | --- | --- | --- | --- | --- | --- | --- |
|  |  | Estimate(95% CI) | P value | Estimate (95% CI) | P value | Estimate(95% CI) | P value | Estimate(95% CI) | P value |
| SII | 444 | 1.07 (0.99 - 1.21) | 0.102 | 1.44 (0.60 - 3.09) | 0.390 | 0.19 (-1.29 - 1.40) | 0.388 | 1.54 (0.66 - 3.32) | 0.31 |
| NLR | 444 | 1.04 (0.99 - 1.16) | 0.156 | 1.50 (0.62 - 3.12) | 0.356 | 0.10 (-0.71 - 1.15) | 0.420 | 1.55 (0.65 - 3.28) | 0.3 |
| PLR | 444 | 1.00 (0.94 - 1.09) | 0.910 | 1.53 (0.65 - 3.23) | 0.314 | 0.01 (-0.54 - 0.64) | 0.952 | 1.53 (0.65 - 3.23) | 0.314 |
| MLR | 444 | 1.04 (0.98 - 1.18) | 0.202 | 1.52 (0.64 - 3.10) | 0.344 | 0.11 (-0.57 - 0.92) | 0.410 | 1.58 (0.65 - 3.28) | 0.300 |
| NPR | 444 | 1.12 (1.01 - 1.35) | 0.032 | 1.26 (0.51 - 2.72) | 0.590 | 0.37 (-3.17 - 2.87) | 0.442 | 1.41 (0.59 - 2.95) | 0.414 |
| SIRI | 444 | 1.15 (1.03 - 1.41) | 0.004 | 1.21 (0.49 - 2.57) | 0.644 | 0.47 (-3.50 - 3.38) | 0.426 | 1.40 (0.58 - 3.05) | 0.43 |
| NMLR | 444 | 1.04 (0.99 - 1.16) | 0.152 | 1.49 (0.61 - 3.13) | 0.356 | 0.11 (-0.73 - 1.18) | 0.416 | 1.55 (0.65 - 3.28) | 0.3 |

ACME, average causal mediation effects (indirect effect); ADE, average direct effects; PE, proportion mediated; TE: total effect；SII, systemic immune - inflammation index; NLR, neutrophil - to - lymphocyte ratio; PLR, platelet - to - lymphocyte ratio; MLR, monocyte - to - lymphocyte ratio; NPR, neutrophil - to - platelet ratio; SIRI, systemic inflammation response index; NMLR, neutrophil - monocyte - to - lymphocyte ratio. The mediation effects presented in the table are estimated based on the fully adjusted model, which accounts for adjustments including gender, age, BMI, acute heart failure, diabetes, shock, SOFA score, antibiotic, insulin, glucocorticoid.

Estimates for the non-diabetic subgroup should be interpreted with caution due to the limited number of non-diabetic population and wide confidence intervals, which indicate statistical instability and reduced power for mediation analysis in this subgroup.


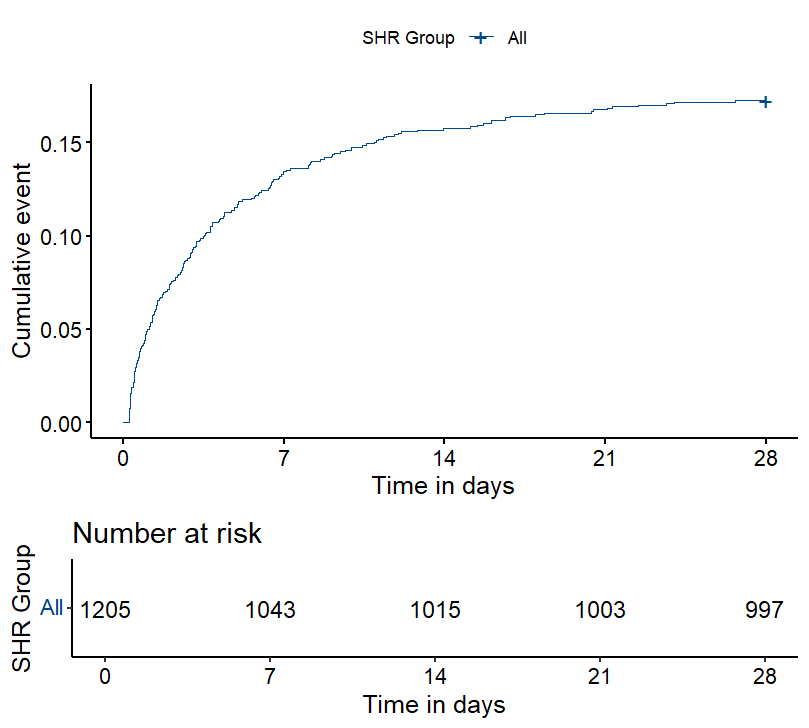


# **Figure S1.** Distribution of time-to-sepsis onset (hours) after intensive care unit admission among 1,205 critically ill patients with heart failure admitted to the intensive care unit at Beth Israel Deaconess Medical Center (Boston, MA, USA) from 2008 to 2022, utilizing data from the MIMIC-IV database. The histogram demonstrates the rationale for selecting a 7-day window as the primary outcome period.


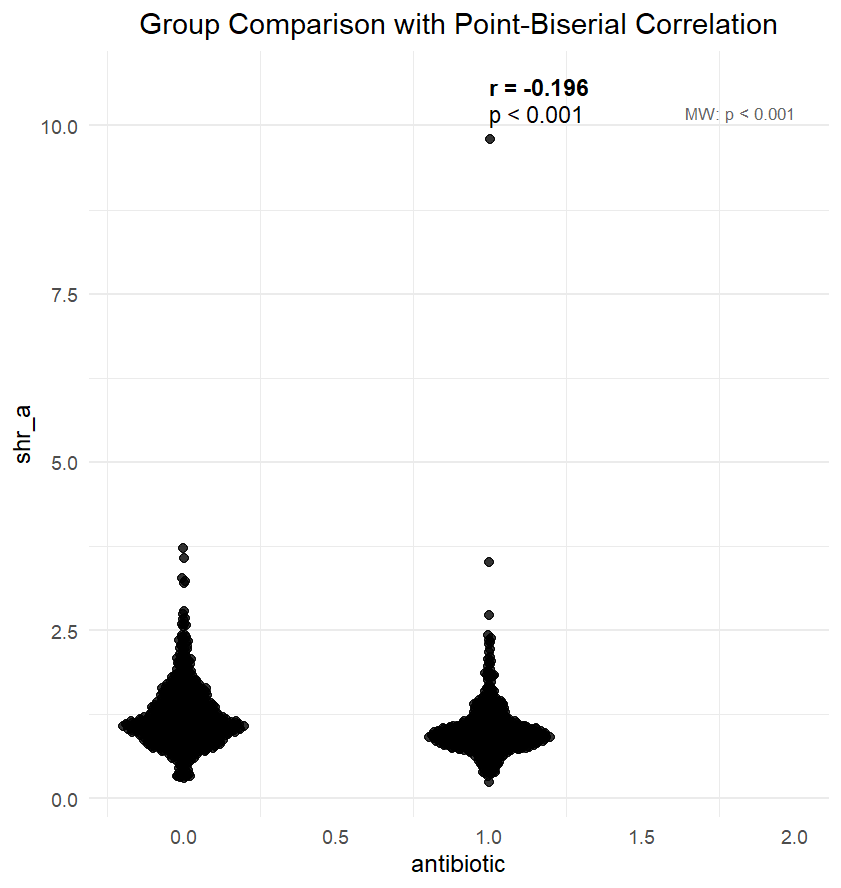


# **Figure S2**. Scatter plot illustrating the association between the Stress Hyperglycemia Ratio and antibiotic use among 1,205 critically ill patients with heart failure admitted to the intensive care unit at Beth Israel Deaconess Medical Center (Boston, MA, USA) from 2008 to 2022, utilizing data from the MIMIC-IV database. The association was assessed using point-biserial correlation due to the binary nature of antibiotic use (yes/no). Antibiotic use was defined as administration during the entire ICU stay. Each data point represents an individual patient, with jitter applied for better visualization.


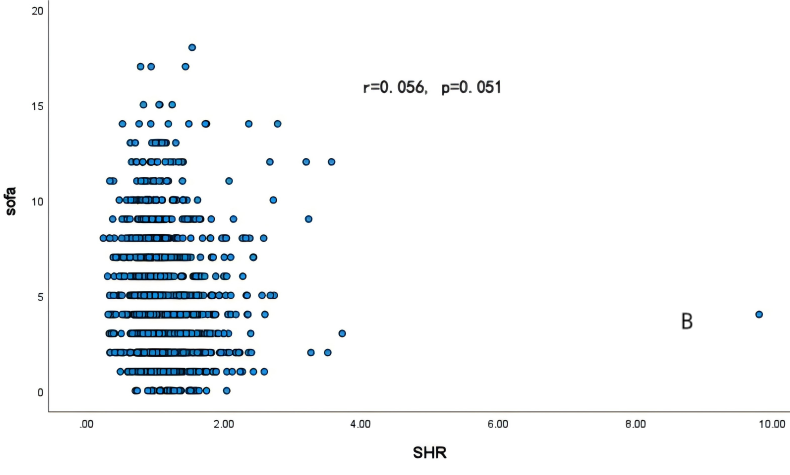


# **Figure S3**. Scatter plot illustrating the association between the Stress Hyperglycemia Ratio and baseline Sequential Organ Failure Assessment score among 1,205 critically ill patients with heart failure admitted to the intensive care unit at Beth Israel Deaconess Medical Center (Boston, MA, USA) from 2008 to 2022, utilizing data from the MIMIC-IV database. The relationship was assessed using Spearman's rank correlation due to the non-normal distribution of SOFA scores.


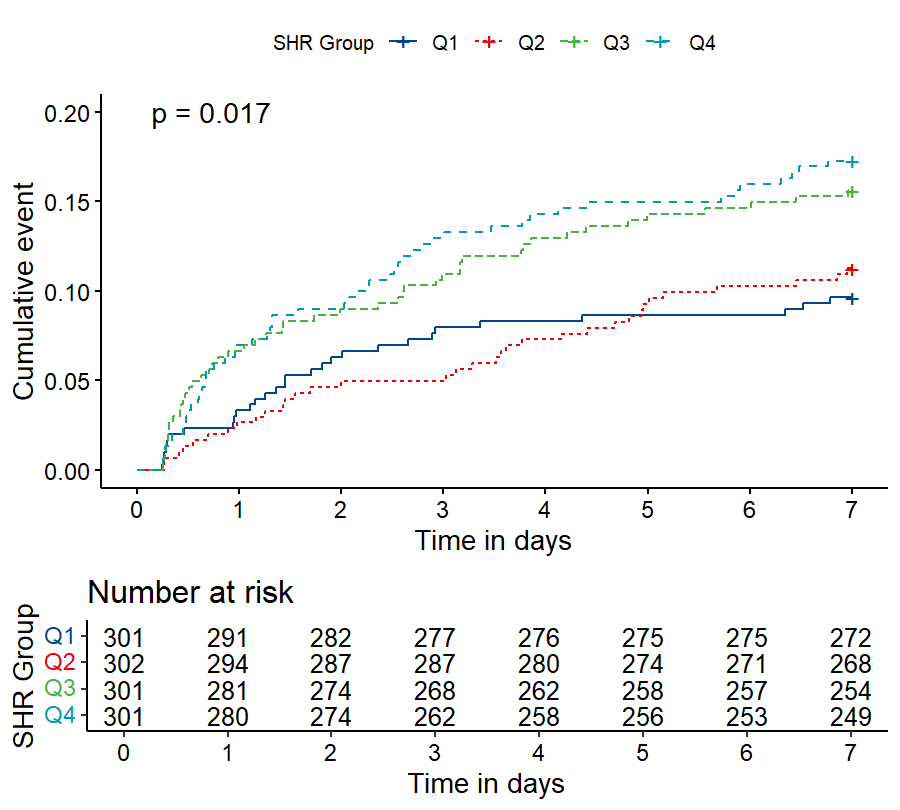


# **Figure S4.** Kaplan–Meier survival curves for 7-day all-cause mortality, stratified by quartiles of the Stress Hyperglycemia Ratio, among 1,205 critically ill patients with heart failure admitted to the intensive care unit at Beth Israel Deaconess Medical Center (Boston, MA, USA) from 2008 to 2022, utilizing data from the MIMIC-IV database. Comparisons were performed using the log-rank test.


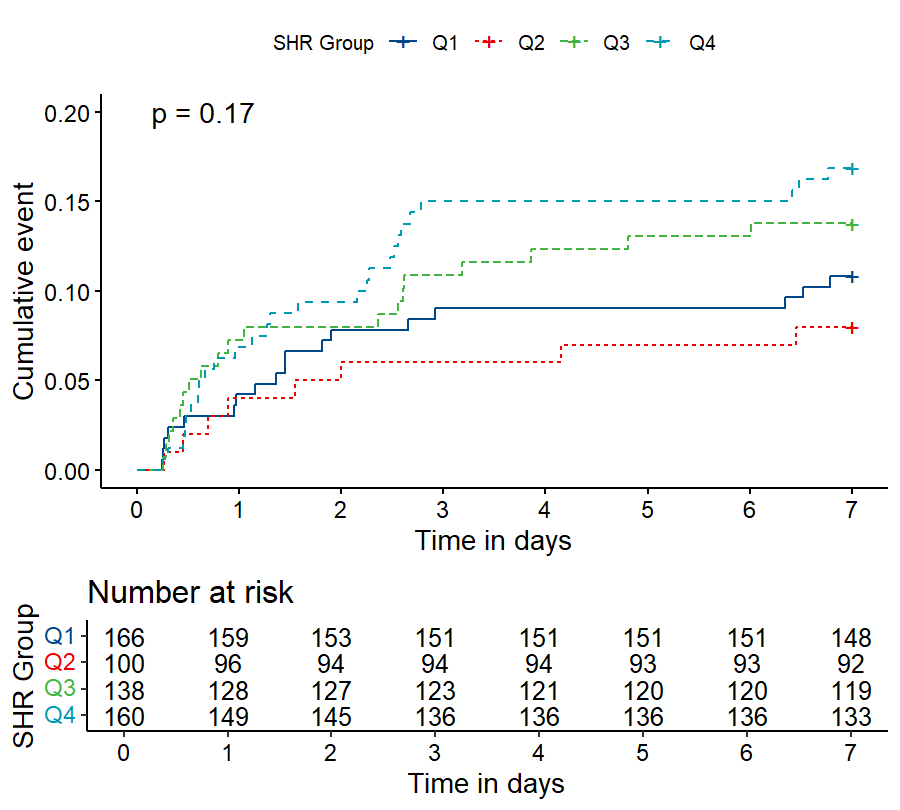


# **Figure S5.** Kaplan–Meier survival curves for 7-day all-cause mortality, stratified by quartiles of the Stress Hyperglycemia Ratio, restricted to the diabetic subgroup (n = 564) of heart failure patients admitted to the intensive care unit at Beth Israel Deaconess Medical Center (Boston, MA, USA) from 2008 to 2022, utilizing data from the MIMIC-IV database.


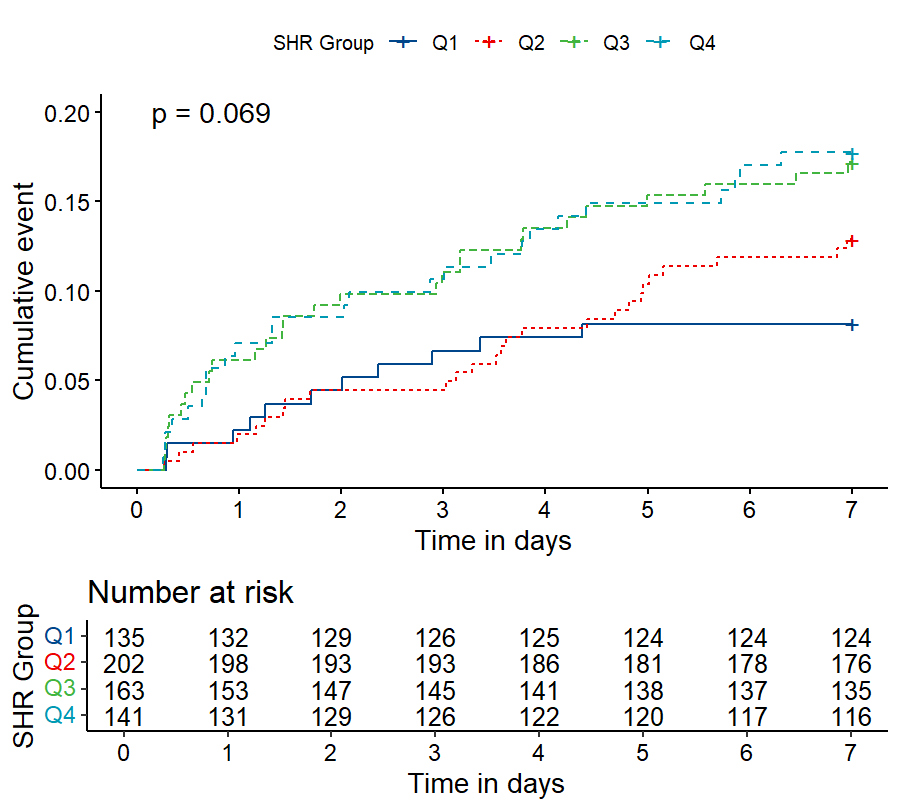


# **Figure S6.** Kaplan–Meier survival curves for 7-day all-cause mortality, stratified by quartiles of the Stress Hyperglycemia Ratio, restricted to the non-diabetic subgroup (n = 641) of heart failure patients admitted to the intensive care unit at Beth Israel Deaconess Medical Center (Boston, MA, USA) from 2008 to 2022, utilizing data from the MIMIC-IV database.


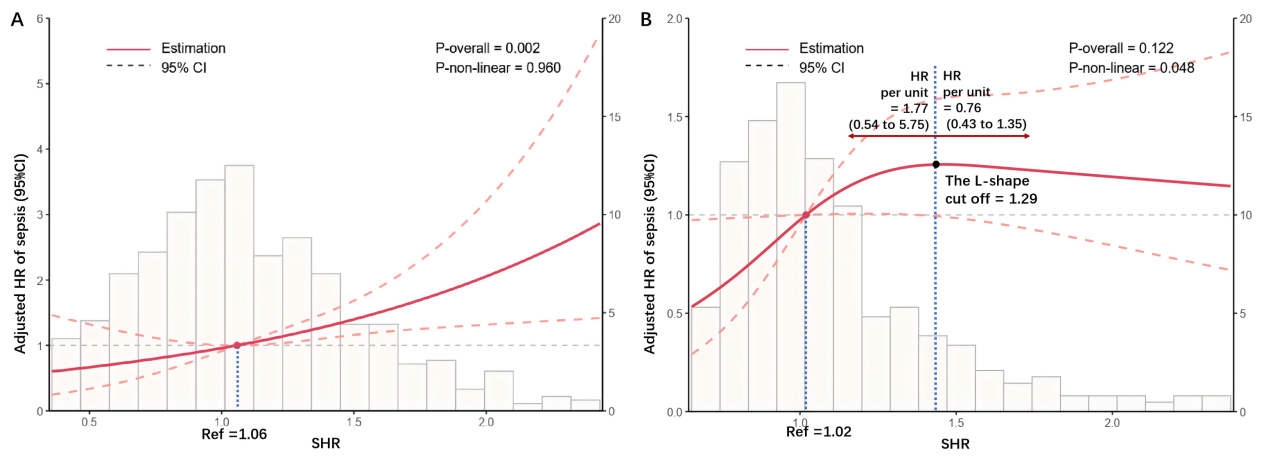


# **Figure S7.** Restricted cubic spline curves depicting the adjusted hazard ratio for the association between the Stress Hyperglycemia Ratio (as a continuous variable) and 7-day sepsis risk, stratified by diabetes status: (A) patients with diabetes (n = 564) and (B) patients without diabetes (n = 641). Analysis was performed among heart failure patients admitted to the intensive care unit at Beth Israel Deaconess Medical Center (Boston, MA, USA) from 2008 to 2022, utilizing data from the MIMIC-IV database. Models were adjusted for gender, age, BMI, acute heart failure, shock, SOFA score, antibiotic use, insulin use, and glucocorticoid use. Curves represent estimated adjusted hazard ratios; shaded ribbons represent 95% confidence intervals. The horizontal dashed line represents a hazard ratio of 1.0.

*HR* hazard ratio, *CI* confidence interval.

# **
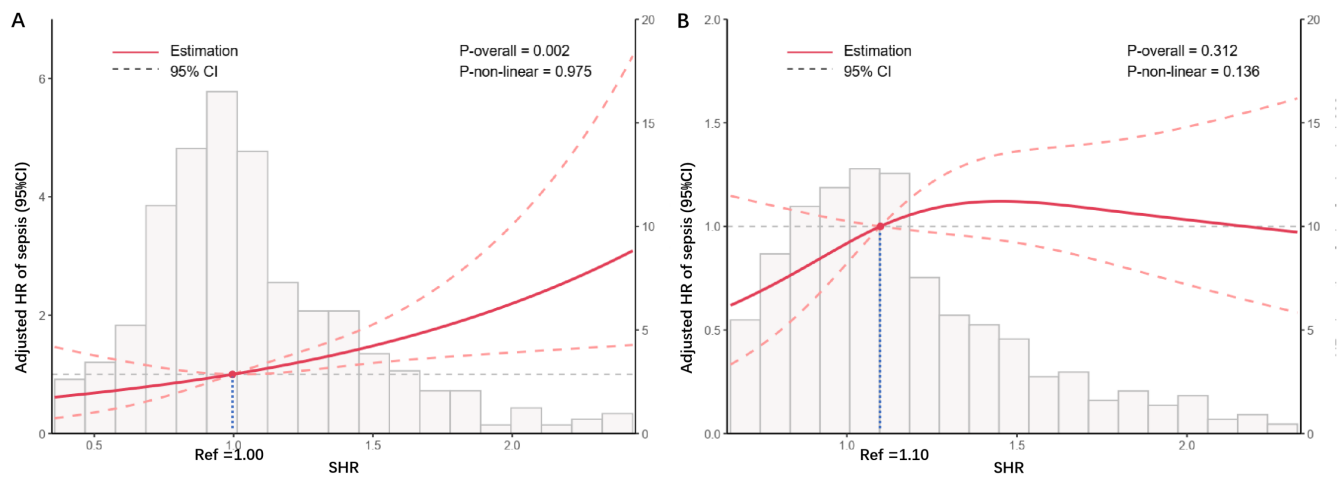
Figure S8.** Restricted cubic spline curves depicting the adjusted hazard ratio for the association between the Stress Hyperglycemia Ratio (as a continuous variable) and 7-day sepsis risk, stratified by baseline insulin use: (A) patients receiving insulin (n = 747) and (B) patients not receiving insulin (n = 458). Analysis was performed among heart failure patients admitted to the intensive care unit at Beth Israel Deaconess Medical Center (Boston, MA, USA) from 2008 to 2022, utilizing data from the MIMIC-IV database. Models were adjusted for gender, age, BMI, acute heart failure, diabetes, shock, SOFA score, antibiotic use, and glucocorticoid use. Curves represent estimated adjusted hazard ratios; shaded ribbons represent 95% confidence intervals. The horizontal dashed line represents a hazard ratio of 1.0.

HR hazard ratio, CI confidence interval.


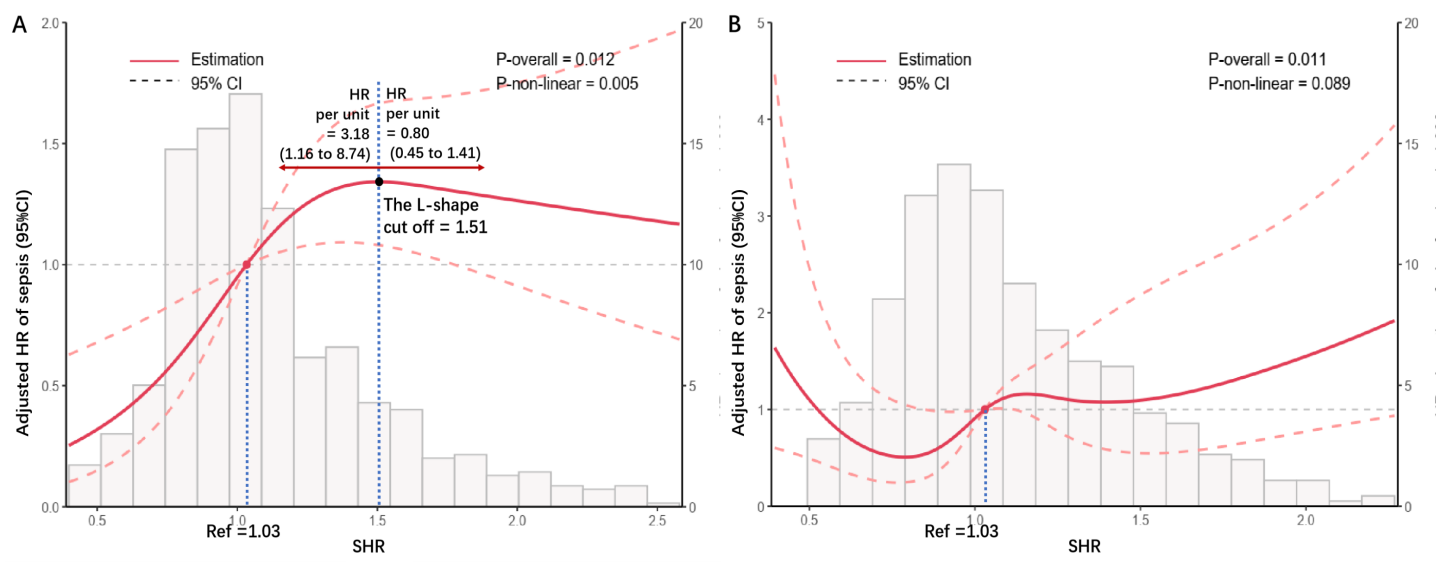


# **Figure S9.** Restricted cubic spline curves depicting the adjusted hazard ratio for the association between the Stress Hyperglycemia Ratio (as a continuous variable) and 7-day sepsis risk, stratified by body mass index: (A) BMI < 30 kg/m² (n = 759) and (B) BMI ≥ 30 kg/m² (n = 446). Analysis was performed among heart failure patients admitted to the intensive care unit at Beth Israel Deaconess Medical Center (Boston, MA, USA) from 2008 to 2022, utilizing data from the MIMIC-IV database. Models were adjusted for gender, age, acute heart failure, diabetes, shock, SOFA score, antibiotic use, insulin use, and glucocorticoid use. Curves represent estimated adjusted hazard ratios; shaded ribbons represent 95% confidence intervals. The horizontal dashed line represents a hazard ratio of 1.0.

HR hazard ratio, CI confidence interval.

1. Estimates for the non-diabetic subgroup should be interpreted with caution due to the limited number of non-diabetic population and wide confidence intervals, which indicate statistical instability and reduced power for mediation analysis in this subgroup. [↑](#footnote-ref-1)
